# Off-target effects of siRNA specific for *GFP*

**DOI:** 10.1186/1471-2199-9-60

**Published:** 2008-06-24

**Authors:** Cordula Tschuch, Angela Schulz, Armin Pscherer, Wiebke Werft, Axel Benner, Agnes Hotz-Wagenblatt, Leticia Serra Barrionuevo, Peter Lichter, Daniel Mertens

**Affiliations:** 1 Division of Molecular Genetics, German Cancer Research Center, Heidelberg, Germany; 2 Division of Biostatistics, German Cancer Research Center, Heidelberg, Germany; 3 Division of Molecular Biophysics, German Cancer Research Center, Heidelberg, Germany; 4 Department of Internal Medicine III, University Hospital, Ulm, Germany

## Abstract

**Background:**

Gene knock down by RNAi is a highly effective approach to silence gene expression in experimental as well as therapeutic settings. However, this widely used methodology entails serious pitfalls, especially concerning specificity of the RNAi molecules.

**Results:**

We tested the most widely used control siRNA directed against *GFP *for off-target effects and found that it deregulates in addition to *GFP *a set of endogenous target genes. The off-target effects were dependent on the amount of *GFP *siRNA transfected and were detected in a variety of cell lines. Since the respective siRNA molecule specific for *GFP *is widely used as negative control for RNAi experiments, we studied the complete set of off-target genes of this molecule by genome-wide expression profiling. The detected modulated mRNAs had target sequences homologous to the siRNA as small as 8 basepairs in size. However, we found no restriction of sequence homology to 3'UTR of target genes.

**Conclusion:**

We can show that even siRNAs without a physiological target have sequence-specific off-target effects in mammalian cells. Furthermore, our analysis defines the off-target genes affected by the siRNA that is commonly used as negative control and directed against *GFP*. Since off-target effects can hardly be avoided, the best strategy is to identify false positives and exclude them from the results. To this end, we provide the set of false positive genes deregulated by the commonly used *GFP *siRNA as a reference resource for future siRNA experiments.

## Background

RNA interference (RNAi) is a powerful method to specifically suppress gene expression and is therefore widely used for experimental as well as therapeutic purposes. Since the initial characterization of RNAi in the nematode *C. elegans *[1], the field of RNAi has expanded remarkably. The essentials of RNAi can be summarized as specific degradation of target mRNA mediated by small, double stranded RNAs [2,3]. Meanwhile, different mechanisms of RNAi were discovered in mammalian cells comprising post-transcriptional gene silencing by siRNAs, transcriptional silencing by siRNAs in the nucleus and the microRNA pathway [4,5]. It has been shown that exogenously introduced siRNA duplexes and endogenously processed miRNA duplexes are taken up by the RNA-induced silencing complex (RISC). The antisense siRNA (guide strand) directs RISC to complementary mRNA, while the second passenger (sense) strand is degraded [6-9].

Recently, the interpretation of RNAi data has become complicated because several studies reported unintended interactions between the silencing molecules and cellular components, so-called off-target effects [10]. Off-target effects include the induction of the antiviral interferon pathway machinery and depend on nucleotide sequence similarity between the siRNA molecule and short motifs in mRNAs of other genes not intended to be knocked-down. A source of sequence-dependent off-target effects in mammals is the high tolerance for mismatches between the siRNA and the complementary target mRNA outside of the first 2–8 bases (seed sequence) of the siRNA [11]. Furthermore, off-target effects can be caused by insertion of the sense siRNA strand into the RISC complex instead of the antisense strand [12]. Finally, off-target effects can occur due to seed-sequence-dependent binding [13].

With these off-target effects in mind, we used a commonly used siRNA sequence directed against *GFP *in a series of microarray expression profiling experiments, in which we knocked down four unrelated candidate suppressor genes (manuscript in preparation). We carefully analyzed the resulting data set and could identify a set of genes commonly deregulated in all experiments that were unrelated except for transfection of the *GFP *siRNA used as negative control. These commonly deregulated genes were subsequently identified as off-target effects of the siRNA against *GFP*. Further investigation by expression profiling revealed the complete set of genes deregulated by the *GFP *siRNA.

## Results

### siRNA specific for *GFP *specifically targets endogenous genes in human cells

**Figure 1:**
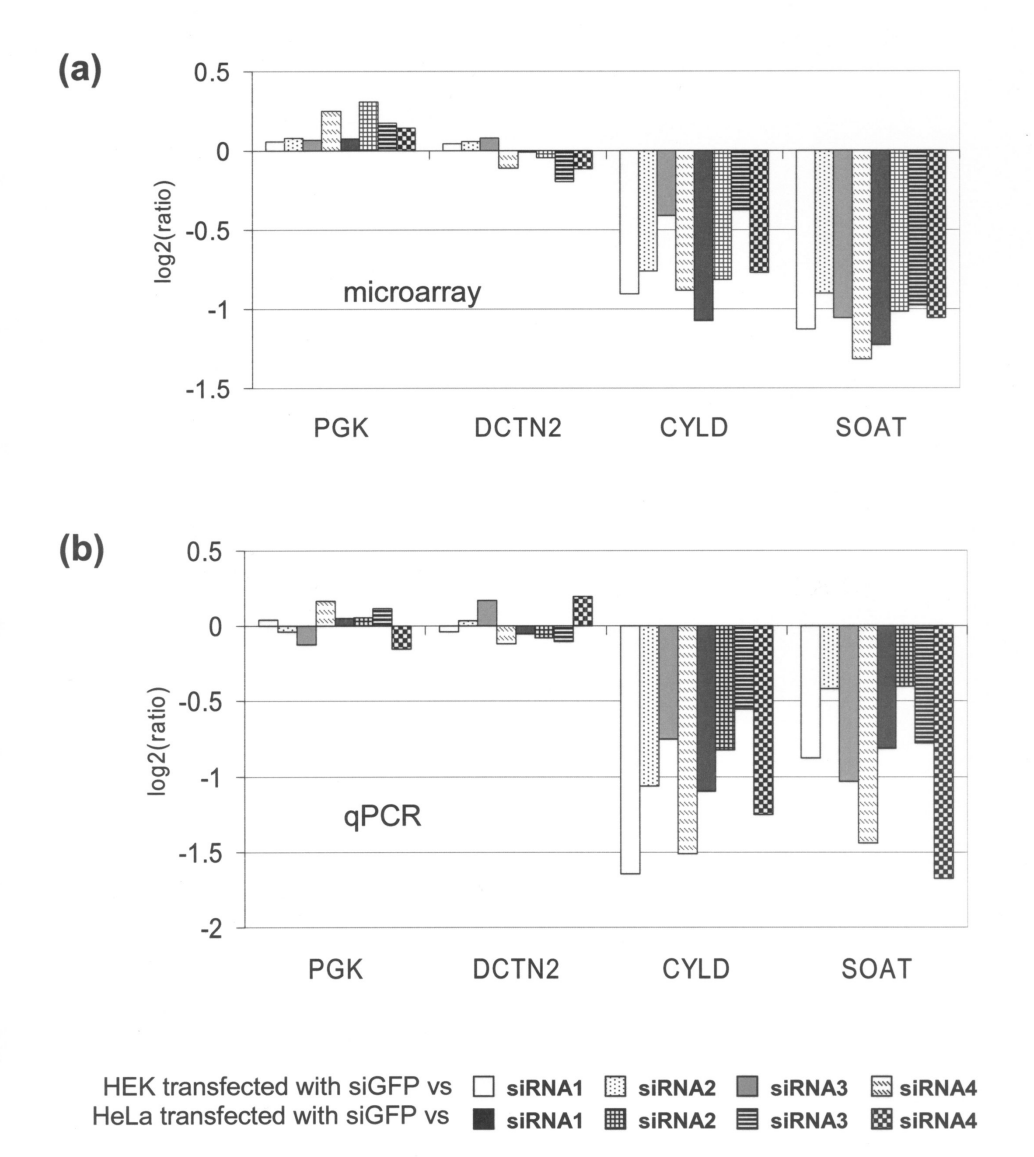
**A siRNA directed against GFP specifically targets endogenous genes in human cells**. (**a**) After transfection of the control siRNA directed against *GFP*, underrepresentation of the off-target genes *CYLD *and *SOAT *was detected by expression-microarrays 48 h post-transfection in HEK and HeLa cells. Normalization was performed as described in Additional file 5. (**b**) Verification of underrepresentation of *CYLD *and *SOAT *in HEK and HeLa cells by Real-Time PCR using the same mRNA as template. Expression of genes was normalized to the median of 2 housekeeping genes (*PGK*, *DCTN2*).

In a set of functional experiments, we used RNAi knock down for the characterization of unrelated candidate suppressor genes in cell lines (manuscript in preparation). Our experimental procedure resulted in efficient knock down of up to 87% in HeLa, 87% in U2OS, 72% in HEK and 68% in EVSAT cells (data not shown). For genome-wide detection of the resulting transcriptional changes, we used oligo-microarrays containing 36,196 oligonucleotides from 25,100 human genes. As negative control, a siRNA against *GFP *was transfected into HEK and HeLa cells and the resulting RNA co-hybridized in all otherwise unrelated array experiments. Due to the issue of off-target effects of siRNA molecules that has been raised recently in the literature, we carefully analyzed the resulting data for genes commonly deregulated in all of the experiments. Indeed, we found a subset of genes to be consistently deregulated, which we postulated to be in fact deregulated by the negative control used in all experiments the siRNA directed against the non-physiological gene *GFP*. Among these off-target genes, *CYLD *and *SOAT *were most consistently and strongest differentially expressed (Figure 1a). Expression levels derived from microarray experiments were normalized as described in Methods or from qPCR were normalized to 2 housekeeping genes that showed constant expression levels in all microarray experiments (*PGK*, *DCTN2*). In comparison to these controls, *CYLD *is down regulated in HEK and HeLa cells between 1.3 and 2.1 fold, and *SOAT *is down regulated between 1.9 and 2.5 fold. This statistically significant deregulation (p < 10^-11^; Table 1) was confirmed by analysis with quantitative PCR (Figure 1b) showing a down regulation of *CYLD *between 1.5 and 3.1 fold (average: 2.2 +/- 0.5) and of *SOAT *between 1.3 and 3.2 fold (average: 2.0 +/- 0.6). Besides of *CYLD *and *SOAT*, we analysed the mRNA levels of a set of off-target genes and found the same effect of down regulation by the *GFP *siRNA in several more genes (see Additional file 1). We analyzed the mRNA sequences of *CYLD *and *SOAT *for sequence homology to the sense and antisense siRNA molecule and found 4 and 2 potential target sites, respectively (data not shown). The control siRNA against *GFP*, which we used, is offered by two different distributors (Table 2 lines 1 & 2). Furthermore a 20 mer siRNA against *GFP *differing in only one basepair is distributed independently (Table 2, line 3). While the off-target genes might differ between these two *GFP *siRNAs, the differences are likely to be small. We characterized the siRNA directed against *GFP *as it is frequently used as negative control in RNAi experiments (Table 2, lines 1 & 2).

**Table 1:** 20 most significantly deregulated genes after transfection of *GFP *siRNA.

**downregulated**				**HELA**			**HEK**		
**#**	**name**	**RefSeq**	**molecular function**	**M**^**X**^	**p**^**§**^	**B**^**S**^	**M**^**X**^	**p**^**§**^	**B**^**S**^

1	**SOAT1**	NM_003101	sterol O-acyltransferase activity	-2.28	8.72E-14	32.76	-0.83	8.18E-07	14.15
2	Q96HQ8	~	unknown	-1.95	8.72E-14	32.87	-1.52	7.71E-12	27.43
3	CASP8	NM_033356	signal transducer activity;caspase activity	-1.56	5.35E-13	30.32	-0.88	6.42E-09	20.37
4	C10orf56	NM_153367	nucleic acid binding;zinc ion binding	-1.62	6.03E-13	30.12	-0.49	1.35E-05	10.51
5	NUDT3	NM_006703	magnesium ion binding;hydrolase activity	-1.45	1.74E-12	28.78	-0.42	6.64E-05	8.35
6	RAB21	NM_014999	nucleotide binding;GTP binding	-1.55	1.89E-12	28.60	-0.76	1.51E-07	16.52
7	HMGCS1	NM_002130	hydroxymethylglutaryl-CoA synthase activity	-1.48	2.73E-12	28.09	-0.45	6.05E-05	8.47
8	PAICS	NM_006452	catalytic activity;ATP binding	-1.37	3.72E-12	27.62	-0.66	3.61E-07	15.39
9	TM16F	NM_001025356	integral to membrane	-1.59	4.96E-12	27.25	-0.45	2.18E-04	6.68
10	GOSR1	NM_004871	v-SNARE activity	-1.47	5.13E-12	27.13	-0.46	6.89E-05	8.29
11	PSF1	NM_021067.3	protein binding	-2.18	5.44E-12	27.01	-1.55	5.47E-09	20.81
12	**CYLD**	NM_015247	NFkB pathway	-1.39	6.75E-12	26.74	-0.60	2.91E-06	12.56
13	CXorf34	NM_024917	methyltransferase activity	-1.68	1.11E-11	26.08	-0.67	1.08E-05	10.82
14	SKP2	NM_005983	protein binding	-1.18	1.80E-11	25.33	-0.74	7.49E-08	17.28
15	IL6ST	NM_002184	interleukin-6 receptor activity;protein binding	-1.20	2.92E-11	24.72	-0.57	3.49E-06	12.27
16	C10orf119	NM_024834	hypothetical protein	-1.14	3.13E-11	24.63	-0.26	5.18E-03	2.29
17	LARS2	NM_015340	leucine-tRNA ligase activity;ATP binding	-1.18	5.29E-11	23.85	-0.49	2.65E-05	9.59
18	NR1D2	NM_005126	transcription factor activity	-1.12	5.63E-11	23.71	-0.32	1.36E-03	4.11
19	ACSL4	NM_022977	long-chain-fatty-acid-CoA ligase activity	-1.22	6.44E-11	23.51	-0.47	7.21E-05	8.21
20	CCD25	NP_060716	unknown	-1.09	1.72E-10	22.18	-0.33	1.67E-03	3.83

**upregulated**				**HELA**			**HEK**		

**#**	**name**	**RefSeq**	**molecular function**	**M**^**X**^	**p**^**§**^	**B**^**S**^	**M**^**X**^	**p**^**§**^	**B**^**S**^

1	ANXA3	NM_005139	diphosphoinositol-polyphosphate phosphatase	2.75	1.13E-15	37.54	0.47	3.89E-05	9.08
2	LOC51149	NM_016175	hypothetical protein	1.29	3.48E-11	24.46	0.29	8.79E-03	1.61
3	DDAH1	NM_012137	hydrolase activity	1.02	1.87E-10	22.07	0.35	5.88E-04	5.27
4	UBP18	NM_017414.2	ubiquitin thiolesterase activity	1.00	1.99E-10	21.99	0.54	4.64E-06	11.91
5	IL15RA	NM_002189	interleukin receptor activity	1.01	2.06E-10	21.94	0.30	2.38E-03	3.34
6	SPHK1	NM_021972	calmodulin binding;transferase activity	1.01	2.16E-10	21.88	0.42	9.44E-05	7.85
7	HIST1H2AC	NM_003512.3	DNA binding	1.27	2.49E-10	21.68	0.54	7.94E-05	8.08
8	MAG1	NM_032717	acyltransferase activity	0.95	2.70E-10	21.53	0.40	1.21E-04	7.53
9	SAT1	NM_002970	acyltransferase activity	1.08	3.21E-10	21.24	0.45	1.46E-04	7.25
10	PTGS2	NM_000963	peroxidase activity;oxidoreductase activity	1.25	4.65E-10	20.78	0.38	3.44E-03	2.84
11	HDHD1A	NM_012080	catalytic activity	0.95	8.31E-10	20.03	0.35	8.84E-04	4.67
12	STAMBPL1	NM_020799	ubiquitin thiolesterase activity	0.80	9.32E-10	19.90	0.30	8.23E-04	4.78
13	PIGL	NM_004278	hydrolase activity	0.83	1.17E-09	19.61	0.36	2.10E-04	6.73
14	CLPB	NM_030813	nucleoside-triphosphatase activity	0.81	2.28E-09	18.74	0.28	3.67E-03	2.75
15	SMPDL3B	NM_014474	hydrolase activity, acting on glycosyl bonds	0.88	2.40E-09	18.67	0.33	1.45E-03	4.02
16	HERC6	NM_017912	ubiquitin-protein ligase activity;GEF activity	0.81	2.69E-09	18.52	0.43	5.32E-05	8.62
17	GLRB	NM_000824	inhibitory glycine receptor,CNS	0.79	4.31E-09	17.90	0.28	3.47E-03	2.82
18	HHLA3	NM_007071	unknown	0.73	5.02E-09	17.71	0.36	1.86E-04	6.89
19	GNPDA1	NM_005471	hydrolase activity	0.63	2.46E-08	15.68	0.36	1.54E-04	7.17
20	PLEKHA7	NM_175058	unknown	0.63	3.29E-08	15.32	0.28	1.97E-03	3.59

^X ^log2 expression ratio, ^§ ^adjusted p value, ^S ^B statistics (for details see Methods and Additional file 5)

**Table 2:** Sequences of the most commonly used control siRNA against *GFP*

**company**	**RNAi molecule**	**target gene**	**sequence 5'-3'**
Ambion	siRNA	GFP/eGFP	*CAA GCU GAC CCU GAA GUU C*TT
Qiagen	siRNA	GFP/eGFP	*CAA GCU GAC CCU GAA GUU C*TT
Dharmacon	siRNA	GFP I	G *CAA GCU GAC CCU GAA GUU C*

### Specific off-target effects recur in different cell lines with different siRNA molecules

**Figure 2:**
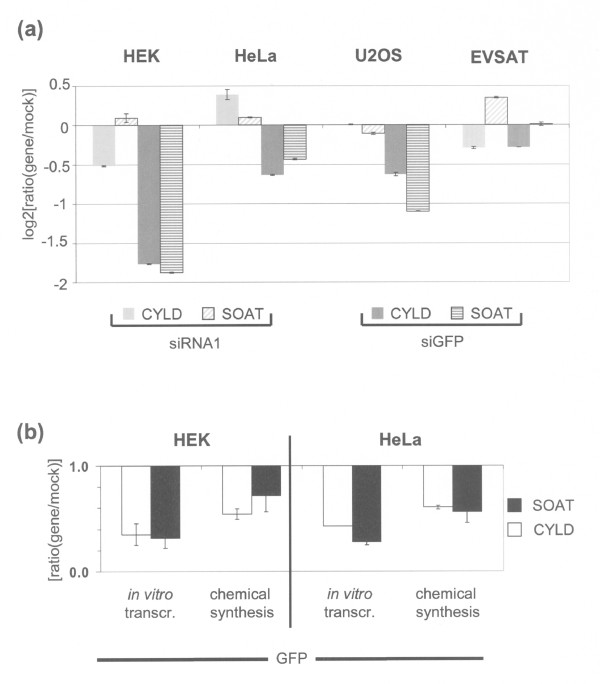
**Off-target effects recur in 3/4 different cell lines and are independent of preparation protocols**. (**a**) After transfection of the siRNA directed against *GFP*, expression of the genes *CYLD *and *SOAT *was measured with qPCR in different cell lines after 48 h. As negative controls, a siRNA directed against a candidate tumour supressor gene (siRNA1) was transfected and a mock transfection was performed. Values are normalized to mock and the median of 2 housekeeping genes. (**b**) 2 different siRNA molecules with the same target sequence but of different methodological origin were transfected, one generated by *in vitro *transcription (see Methods) and a second siRNA was obtained by oligonucleotide synthesis (Table 1). After transfection, the expression of *CYLD *and *SOAT *was measured in HEK and HeLa cells and normalized expression after mock transfection and the median of 2 housekeeping genes (*PGK, DCTN2*).

**Figure 3:**
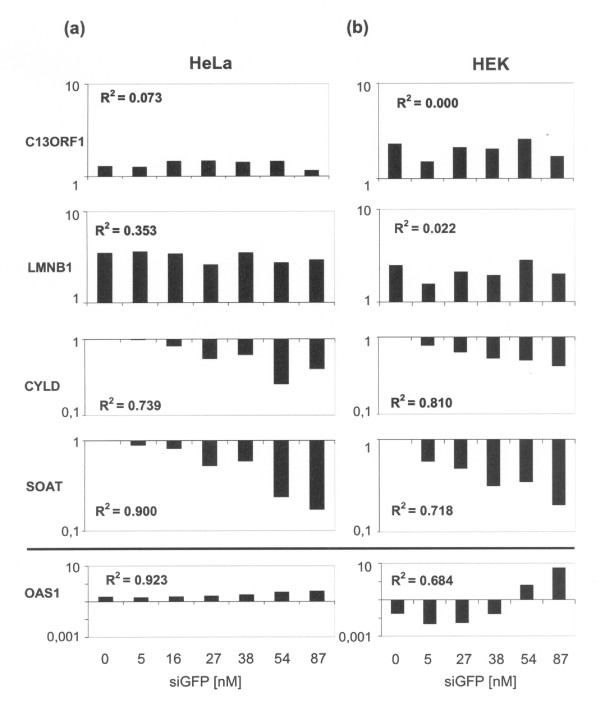
**Degree of off-target knock down of CYLD and SOAT correlates with the concentration of the transfected siRNA**. (a) Different concentrations of the siRNA directed against *GFP *were transfected in HeLa and (b) HEK cells. Expression of specific off-target genes (*CYLD, SOAT*), non-target genes used as negative control (*LMNB1, C13ORF1*) and genes unspecifically upregulated by antiviral cellular response (*OAS1*) were measured by qPCR 48 h post-transfection. Expression values were normalized to the average of 2 housekeeping genes (*PGK*, *DCTN2*). Correlation coefficients between the amount of siRNA transfected and the corresponding mRNA levels of the genes measured are given.

We found specific off-target deregulation of *CYLD *and *SOAT *by *GFP *siRNA in 3/4 cell lines derived from different tissues as compared to mock transfections (Figure 2a). Only EVSAT cells did not show a reduced expression of *CYLD *and *SOAT*, which is probably due to the lower transfection efficiency (data not shown). In order to exclude that the observed off-target effects are due to our preparation of the siRNA, we compared its effects to chemically synthesized siRNA directed against *GFP*. While the sequences of these two molecules are identical, we used *in vitro *transcription to produce siRNA, while the commercially available siRNA was produced by oligonucleotide synthesis. However, transfection of either of these siRNAs into HEK and HeLa cells resulted in similar decrease of expression of *CYLD *and *SOAT *as compared to the mock transfection (Figure 2b). We excluded that the observed effects are due to induction of unspecific interferon response by measuring expression of the *OAS1 *gene [14,15], which was not induced after transfection of 38 nM siRNA molecule (Figure 3). Therefore, the off-target deregulation of *CYLD *and *SOAT *is not dependent on the methodological origin of the RNAi molecule.

### Distinct off-target effects depend on the concentration of *GFP *siRNA

In order to assess specificity of the observed off-target effect, we transfected increasing amounts of the siRNA directed against *GFP *and measured expression of *CYLD *and *SOAT *as off-target genes and of *C13ORF1 *and *LMNB1 *as non-regulated, negative controls using qPCR (Figure 3a, b). In addition, in order to detect induction of unspecific antiviral interferon response,*OAS1 *was measured in transfected cells. As expected, increasing amounts of transfected *GFP *siRNA did not have any influence on the expression level of the genes *C13ORF1 *and *LMNB1 *that were used as negative controls. In contrast, the mRNA levels of *CYLD *and *SOAT *decreased with increasing amounts of transfected *GFP *siRNA, pointing to targeted down regulation of these genes by the *GFP *siRNA both in HeLa (Figure 3a) and HEK cells (Figure 3b). Furthermore, knock down of *CYLD *and *SOAT *mRNA correlated with the concentration of the transfected siRNA (0.718<R^2^<0.900). Thus, the off-target effect for *CYLD *and *SOAT *is dependent on the amount of transfected siRNA against *GFP *in HEK and HeLa cells, supporting selectivity of the observed effect. In contrast, levels of *C13ORF1 *and *LMNB1 *mRNA did not correlate with the amount of transfected *GFP *siRNA (0.000<R^2^<0.353). Transfection of increasing amounts of siRNA molecules induced the non-specific antiviral interferon response in HEK cells as reported previously [16], while Hela cells were unable to mount this response, as expected [17]. However, the interferon response in HEK cells was only induced using 500 ng (54 nM) of transfected siRNA, a concentration at least one order of magnitude higher than the siRNA concentration at which we observed the sequence-specific knockdown of *CYLD *and *SOAT*.

### mRNAs deregulated by *GFP *siRNA cannot be functionally attributed to a single cellular process

In general, siRNA molecules can impact on target mRNAs via two pathways: while imperfect matching mRNA molecules are impeded mostly in their translation, perfect matches lead to mRNA degradation [18]. To determine the mechanism of how the *GFP *siRNA affects the mRNA transcript and also to define the set of target genes of the *GFP *siRNA, we transfected HeLa and HEK cells with the siRNA directed against *GFP *and analyzed transcriptional changes on an additional second set of expression microarrays. Overall, 397 genes were significantly deregulated compared to the mock transfection (Table 1, for a complete list see Additional file 2). Intriguingly, a similar number of genes was significantly over- (190, 48%) and underrepresented (207, 52%), which means that a large number of transcripts changed their levels due to secondary indirect effects. Next, we wanted to find out whether the majority of mRNAs were deregulated indirectly due to direct modulation of a single cellular pathway or transcription factor. As can be seen from the function of deregulated genes (Table 1, Additional file 2), the deregulated mRNAs belong to a diverse set of cellular pathways. This excludes that the off-target effects that we see are due to deregulation of a single central biochemical pathway.

### Down regulation of off-target genes correlates with very short stretches of perfect sequence homology

**Figure 4:**
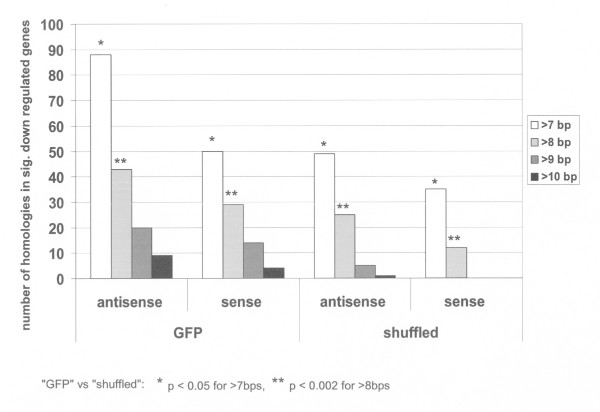
**mRNAs deregulated by GFP siRNA contain short sequences perfectly matched to the sense or antisense strand of GFP siRNA**. In order to shed light on the molecular mechanism of sequence-specific off-target effects, we analyzed the mRNAs modulated after transfection of *GFP *siRNA for sequence homology to the transfected siRNA molecules. The number of perfect matches with a homology length of at least 8 bp was significantly higher using the proper *GFP *siRNA sequences as compared to the shuffled siRNA sequences.

In order to find common motifs in the sequences of target genes deregulated by the *GFP *siRNA, we looked at the second set of expression profiling experiments that was dedicated only to the characterization of off-target-effects of *GFP *siRNA. To this end, we analyzed the sequences of 207 genes that were significantly down regulated after transfection of *GFP *siRNA (see Additional file 2) for sequence homology to the *GFP *siRNA sense and antisense molecules (Figure 4). Since upregulation of genes is probably not due to direct targeting by the siRNA via sequence homology but rather likely a secondary effect, we focused in our statistical analysis on genes that were down regulated by the *GFP *siRNA. To this end, we aligned the sequences of the 207 down regulated mRNAs to the *GFP *siRNA sequence in order to identify likely direct targets. In our set of 207 significantly underrepresented mRNAs, we found 50 mRNAs with an 8 mer homology to the sense and 88 to the antisense *GFP *siRNA (116/207 down regulated genes, 56%, Figure 4). Of these mRNAs, 22 showed homology for both siRNA molecules albeit at different sequence positions. In order to find out whether this concomitant binding of both sense and antisense siRNA molecules would result in a more pronounced down regulation of mRNA molecules with homologies for both siRNAs, we tested whether these mRNAs would be underrepresented to a greater extent in our microarray analyses. However, no significant down regulation of these 22 mRNAs could be detected compared to the remaining mRNAs with only one sequence homology. This could of course be due to the limitation of scoring only perfect matches and disregarding homologies with single mismatches, which reportedly plays a major role in the siRNA silencing mechanism [19]. In order to assess whether the homologies detected in our set of underrepresented mRNAs could also occur by chance, we performed the same homology searches with shuffled *GFP *sense and antisense sequences. Compared to the proper sequences, we found significantly less homologies using these shuffled *GFP *sequences (p < 0.05 for matches > 7 bp and p < 0.002 for matches >8 bp; Figure 4).

In order to exclude a bias due to composition of dinucleotides in the siRNA sequences, we additionally shuffled the *GFP *siRNA sequence while preserving its dinucleotide composition and also found less sequence homologies as compared to the proper *GFP *siRNA sequence, even though the difference was not as pronounced (see Additional file 3). Therefore, preservation of dinucleotide composition seems to retain a low level of homology to the genes deregulated by the *GFP *siRNA. Also we searched for sequence homologies of the proper *GFP *siRNA sequences in a set of mRNA sequences that were not deregulated in our experiments or that were randomly picked. With increasing length of homology, relatively more homologies were found with the proper, non-shuffled *GFP *siRNA in the set of actually deregulated genes as compared to all negative controls (see Additional file 3).

Next we wanted to determine whether there was a clustering of sequence homologies in specific regions of the siRNA or mRNA molecules. In contrast to previous findings [20], we found no enrichment of sequence homology of the siRNA sequences neither in the 3'UTR of deregulated mRNA sequences nor anywhere else in the transcripts. Rather, the siRNA of *GFP *shows sequence homology to all parts of candidate target mRNAs (data not shown). Also, we did not find enrichment of sequence homology in the 5'seed region of the siRNA molecule.

## Discussion

RNA interference is a potent method of gene silencing that has rapidly become important over the past years and that is now widely used for experimental as well as therapeutic purposes. However, the method harbours several pitfalls, one of them being the artefactual dysregulation of non-target genes. This effect can either be due to i) induction of the interferon response in mammalian cells after transfection of RNAi molecules or ii) result from the unintended targeting of genes that have only low level of sequence homology to the RNAi molecule. While the former unwanted effect is avoidable, the latter artefact cannot be predicted. This means that even siRNAs with no predicted physiological target sequences – which are often used as negative controls for RNAi experiments – will have specific off-target effects, and these are thought to be caused by sequence similarity of very short seed sequences [13,20]. This has prompted calls for rigorous standards in siRNA experiments, especially in large-scale screens [10]. Here, we show that siRNA molecules that are commercially distributed and used widely as negative controls actually target endogenous genes with important roles in several pathways, even though there are only very small regions of sequence homology between the siRNA and the mRNA molecules.

We used siRNA directed against *GFP *as negative control in a series of unrelated knock down experiments that were analyzed by expression microarrays (manuscript in preparation). Due to the ongoing discussion concerning the specificity of RNAi molecules, we rigorously screened the data and paid special attention to genes that were commonly deregulated in all of these expression profiling experiments, which were unrelated except for the use of *GFP *siRNA as negative control. Indeed, we detected strong dysregulation of the genes *CYLD *and *SOAT *in all of these experiments (Figure 1a) and in various cell lines (Figure 2a). Only EVSAT cells did not show a reduced expression of *CYLD *and *SOAT *after transfection of *GFP *siRNA. This is probably due to the lower transfection efficiency of EVSAT cells compared to HEK, HeLa or U2OS cells. Furthermore, we show that the strong dysregulation of *CYLD *and *SOAT *is independent of the synthetic origin of the siRNA molecules (Figure 2b). Therefore, the commonly used siRNA directed against *GFP *has sequence-dependent off-target effects in human cells, with the genes *CYLD *and *SOAT *showing most pronounced deregulation.

Are these effects sequence-dependent or caused by sequence-independent effects? In general, unwanted effects like activation of the interferon response are more likely to occur when high concentrations of siRNA are used. How low the concentration of transfected dsRNA molecules must be to prevent unspecific effects is controversial: While there are reports that siRNA concentrations of ≤ 20 nM usually do not lead to induction of the interferon response [21], others have detected unspecific effects using siRNA concentrations as low as 10 nM [15]. In this study, we transfected increasing amounts of siRNA directed against *GFP *(Figure 3). In HEK as well as in HeLa cells, we could show that off-target knock down of *CYLD *and *SOAT *correlates with the concentration of the transfected siRNA. Already a concentration of 5 nM siRNA showed the down regulation of *CYLD *and *SOAT *as off-target genes. At the same time, the interferon response is activated only after transfection of more than 54 nM siRNA, pointing to a directed targeting of *CYLD *and *SOAT *and not a down regulation which is concomitant to the interferon response mechanism. In order to shed light on this gene-specific mechanism, we performed a genome-wide screen to identify all mRNA transcripts deregulated by the *GFP *siRNA. Intriguingly, we found a similar number of mRNAs overrepresented as mRNAs underrepresented after transfection of *GFP *siRNA, which means that at least half of the deregulation that we detected is due to secondary effects. An earlier study could not detect off-target effects for a siRNA directed against *GFP *[22]. In the light of the recent progress of the field and several reports on off-target effects, this finding seems highly unlikely since the majority of siRNA molecules, even those with a non-physiological target, will be able to pair to a set of mRNAs with partial homology. In fact, we reproducibly found a large number of genes specifically deregulated after transfection of *GFP *siRNA.

In order to shed light on the mechanism of action of the *GFP *siRNA, we looked for sequence homologies between the *GFP *siRNA and the deregulated mRNAs. In our set of mRNAs that were underrepresented after transfection of *GFP *siRNA, we found significantly more hits with the proper *GFP *siRNA sequence as compared to the shuffled siRNA sequences for sequence homologies of more than 7 bp length. Of the 207 genes down modulated, we found perfect matches of more than 7 bp in 116 (56 %) of the down modulated genes. 22 mRNAs showed homology to both the sense strand as well as the antisense strand of the *GFP *siRNA.

In contrast to the current model where binding of several siRNA molecules results in more consistent down modulation of the mRNA [23], we did not find these 22 mRNAs to be more underrepresented as compared to the mRNAs with homologies to one of the siRNA molecules only. This could be due to our scoring of perfect, non-mismatch homologies only due to computational limitations [10]. Interestingly, in our system both strands of the transfected double-stranded siRNA duplex show a significant number of homologies to the underrepresented mRNAs as compared to the shuffled control sequences. While there is a bias towards usage of the antisense strand as required, both strands of the siRNA are loaded into the RISC complex. This could be reduced by proper design of the *GFP *siRNA sequence which would prevent loading of both sense and antisense molecules into the RISC complex [24]. Down regulation of the remaining 91 (44 %) transcripts without perfect matches to the siRNA sequences is probably due to pairing with partial mismatches, whose *in-silico *prediction is almost impossible [10]. Additionally, a proportion of these remaining 44 % of deregulated mRNAs without perfect match to *GFP *siRNA is also likely to be modulated indirectly as secondary effect, just as the 190 mRNAs that are up regulated after transfection of the siRNAs. While the indirect target mRNAs may be of little interest for the molecular mechanism, these secondary effects will be just as confounding in experiments using *GFP *siRNA as the direct effects. According to our data, possibly as many as 14 % of sequence-specific off-target effects could be avoided by design in order to exclude one of the two strands of the transfected siRNA from the RISC complex [9,8]. Very recently, several vendors introduced a new generation of chemically modified siRNAs that are supposed to ensure loading of the siRNA antisense strand into the RISC complex only, thereby reducing off-target effects.

With increasing length of homology, relatively more hits were scored for the proper, non-shuffled *GFP *siRNA in the set of actually deregulated genes as compared to all negative controls (Figure 4; Additional file 3). It is remarkable that preservation of dinucleotides while shuffling of *GFP *siRNA sequence results in a larger number of sequence homologies in deregulated genes. It seems that the dinucleotides in the *GFP *siRNA sequence have a background homology in human genes, which is abrogated by complete shuffling of the dinucleotide composition.

Interestingly, in contrast to previous findings [13,20], homologous sequences were not clustered in the 5' seed sequence of the *GFP *siRNA and they were also not clustered in the 3'UTR of target mRNAs or any other region of the mRNA. In Jackson *et al*., [13], the authors performed transcriptome-wide time course analyses to identify off-target mRNA transcripts, which allowed limiting the number of primary off-target genes to 9 only. Within these 9 genes, the authors could search for regions with only partial and very small homology (e.g. 5/11 bp identities). In contrast, we could only analyze a single time point and found 207 down modulated genes, which is why we had to restrict our homology search to perfect matches only, and this could be a confounding factor.

How to handle sequence-specific off-target effects in siRNA experiments? Proper design of the sequence of siRNAs using in-silico target gene prediction will not avoid down regulation of off-target mRNA transcripts as shown here and discussed previously [10]. The aim should therefore not be to avoid these effects but rather to identify false positives and exclude them from the set of deregulated mRNAs. In order to help identify false positive target genes that really are off-target genes in future knock-down experiments, we here present the endogenous genes that are down regulated by one of the most commonly used control siRNA directed against *GFP*. Another strategy to identify off-target genes arising from the control siRNA is to use several different control siRNAs which are directed against different sites in non-endogenous genes like green fluorescent protein and Luciferase in addition to a mock transfected control. Also, the sequence of the siRNA directed against the gene of interest could be scrambled and the resulting molecule be used as a negative control. However, the most straightforward approach is to employ several different siRNA molecules to target the mRNA of interest and identify true targets by their property of being knocked-down by all of these siRNAs [25]. The most comprehensive approach of this kind would be to use esiRNAs, which should then be produced from the whole target mRNA sequence. In this case, while all of the resulting esiRNA molecules would target the single mRNA of interest, the off-target effects of the single esiRNA that are present only at very low concentrations compared to all other esiRNA molecules would be minimal and probably below detection level. Furthermore, the transfection of shRNA plasmids by lenti- or retroviruses is an option for reducing off-target effects, since the level of stable shRNA expression is comparatively modest which results in minimal off-target effects. A second more laborious approach is to rescue the observed siRNA phenotype by transfecting a recombinant cDNA that is mutated at the siRNA target sequence(s) and thus rendered non-responsive to the siRNA. Alternatively, after using a siRNA directed against the 3'UTR of a certain gene, rescue of expression can be achieved by expression of the mRNA lacking its normal 3'UTR sequence. Ideally, both approaches of i) the usage of several siRNA molecules and ii) rescue of the phenotype by non-responsive cDNA plasmids should be combined in experimental strategies. Only these precautions will allow to definitely exclude that the observed phenotype of the siRNA knockdown is due to unwanted artefactual off-target effects.

## Conclusion

Our analysis shows that even a siRNA frequently used as negative control that is directed against exogenous *GFP *has off-target effects in mammalian cells. This siRNA is distributed by several companies and widely used for RNAi experiments. Since off-target effects seemingly cannot be avoided, the best strategy is to identify these off-target genes as false positives and exclude them from the set of deregulated mRNAs. In addition to proving that a siRNA used as a negative control has sequence specific off target effects, we also provide a list of deregulated target genes of the exogenous *GFP *siRNA that can be used as reference for future RNAi experiments.

## Methods

### Cell lines

HEK293, HeLa, U2OS and EVSAT cells were obtained from ATCC and were maintained in DMEM containing 10% fetal bovine serum (FBS) and 1% antibiotics at 37°C in a humidified atmosphere containing 5% CO_2_. Cells were passaged every 3–4 days.

### siRNA design and synthesis via *in vitro *transcription

For the design of effective siRNA target sequences, a siRNA design tool was used [26] and siRNA target sequences were chosen according to published criteria [27,9,8].

For synthesis of siRNAs via *in vitro *transcription, the Silencer™ siRNA Construction Kit (Ambion, Austin, USA) was used with modifications [28].

Chemically synthesized *GFP *siRNA was obtained from Ambion (Ambion, Austin, USA).

### siRNA transfection and RNA isolation

Transient transfection of cells was performed with Effectene transfection reagent (Qiagen, Hilden, Germany) according to the manufacturer's protocol. For transfection in a 48-well format, we used reagents suggested for a 24-well transfection to increase efficiency. In order to exclude artefacts due to this change in procedure, control experiments were treated in exactly the same way. 6–8 h after transfection, medium was replaced. Afterwards, cells were incubated under normal growth conditions and harvested 48 h after transfection. If not mentioned differently, siRNAs were transfected with a concentration of 38 nM, which corresponds to 350 ng siRNA employed per 48-well reaction.

RNA was isolated from cell pellets frozen at -80°C using the Absolutely RNA Micro Prep Kit (Stratagene, La Jolla, USA). For array experiments, cell pellets were resuspended in Trizol (Invitrogen, Karlsruhe, Germany), total RNA extracted according to the protocol and further purified on Rneasy Mini spin columns (Qiagen, Hilden, Germany).

### Transcriptome amplification and labelling for microarray experiments

Amplification of sample RNA was done according to the TAcKLE protocol with modifications [29]. Briefly, 2 μg total RNA was employed in first- and second-strand cDNA synthesis. Double-stranded cDNA was extracted, dissolved in 10 μl nuclease-free water and employed for *in vitro *transcription using RiboMAX Large Scale RNA Production System T7 (Promega, Karlsruhe, Germany) according to the manufacturer's recommendations in 40 μl reaction volume for 12 h. Samples were labelled with fluorochrome as described previously [29].

### Hybridization of oligo-microarrays

A set of 36,196 gene-specific 70 mer oligonucleotides (Human Oligo Set 4.0; Operon, Cologne, Germany) was printed in unicates on glass slides coated with epoxy-silane (Schott Nexterion, Jena, Germany).

After completion of the labelling reactions, control and sample cDNA was combined and purified on Microcon YM-30 filter columns (Millipore, Schwalbach, Germany) as suggested by the manufacturer. For blocking of repetitive sequence elements, 25 μg Cot-1 DNA (Roche Diagnostics, Mannheim, Germany), 25 μg poly-A RNA (Sigma-Aldrich, Munich, Germany) and 75 μg yeast tRNA (Sigma-Aldrich, Munich, Germany) were added before the final washing step. Just prior to hybridization, slides were washed for 2 min in 0.2% SDS (w/v), 2 min in ddH_2_O at RT and 2 min in boiling ddH_2_O (95°C), followed by 1 min centrifugation at 1000 rpm. Purified dye-labeled cDNA was mixed with 140 μl Ultra-Hyb hybridization buffer (Ambion, Austin, USA), agitated for 60 min at 60°C and for 10 min at 70°C and subsequently applied to pre-heated (60°C) microarrays mounted in a GeneTAC Hybridization Station (Genomic Solutions, Ann Arbor, USA). Hybridization reactions were performed for 40 h at 42°C with gentle agitation. Thereafter, arrays were automatically washed at 36°C with 0.5× SSC, 0.1% (w/v) SDS for 5 min; 0.05× SSC, 0.1% (w/v) SDS for 3 min; 0.05× SSC for 20s and 0.05× SSC, 0.1% (w/v) Tween20 for 20s. Flow time was set to 40s. Immediately after completion of the final wash step, arrays were collected, immersed in 0.05× SSC, 0.1% (w/v) Tween20 for transportation and dried by centrifugation in 50 ml Falcon tubes for 3 min at 2500 rpm.

### Data acquisition of microarray experiments, quality control and normalization

Hybridized microarrays were scanned at 5 μm resolution and variable PMT voltage to obtain maximal signal intensities with < 0.1% probe saturation, a count ratio of 0.8–1.2 (Cy5/Cy3) and maximal congruence of histogram curves using a GenePix 4000B microarray scanner (Axon Instruments, Union City, USA). Data summaries of the spot intensities were provided by GenePix Pro, Version 5.1 (Axon Instruments, Union City, USA) using local background correction and including mean and the median pixel intensities at each wavelength for both feature and background pixels.

For quality control of individual spots, boolean quality flag information was given by GenePix Pro pre-processing. Additionally, spots were dismissed if either the feature to background ratio of the median intensity values at both wavelengths were less than 1.5 or the absolute log2 ratio of the mean and median feature intensity varied by more than 0.25 for at least one wavelength. For all statistical analyzes, the log2 ratio of the median spot feature intensity at wavelength 635 (Cy5) and 532 (Cy3) was used.

Normalization of spot intensities was performed by variance stabilizing transformation, which calibrates for sample-to-sample variations through shifting and scaling and transforms the intensities to a scale where the variance is approximately independent of the mean intensities [30]. The procedure provides normalized intensities on a log2 scale. Log2 expression ratios (M-values) were obtained by subtracting the transformed intensities at both scanned wavelengths.  In detail, normalization was performed as described in Additional file 5.

The 20 most deregulated genes after transfection of siRNA directed against *GFP *are listed in Table 1. A complete list of deregulated genes is available (see Additional file 2). Deregulated genes in both tables are sorted for the adjusted p value of experiments performed in HeLa cells. Information about molecular function is based on GO terms. The M value depicts the degree of deregulation, while the B value is a measure of significance of the deregulation. Raw and normalized data are deposited in the Gene Expression Omnibus database (accession No. GSE8680) [31].

### Real-time PCR quantification

cDNA templates were generated from the corresponding mRNA using SuperScript II and anchored oligo-d(T)_20 _primer (Invitrogen, Karlsruhe, Germany). For amplification and quantification, SYBR Green ROX Mix (Abgene, Epsome, UK) was used. To prevent amplification of genomic DNA, all amplicons were designed to span exon-exon boundaries and were tested using genomic DNA as negative control. Real-Time PCR was performed in a 7900RT Fast Real-Time PCR System (Applied Biosystems, Forster City, USA) with the following settings: initial denaturation at 95°C for 15 min, amplification and quantification at 95°C, 15s; 60°C, 10s; 72°C, 60s for 40 cycles with single fluorescence measurement. Products were analyzed by melting-curve analysis (60°C to 95°C with 0.1K/s continuous fluorescence measurements). Calculation of efficiency and relative quantification versus non-regulated standard genes (*PGK*, *DCTN2*) were performed as described previously [32,33]. Error bars depict standard deviation of duplicate qPCR reactions. Additional file 4 shows used qPCR primer sequences.

### Detection of sequence homologies between deregulated mRNAs and *GFP *siRNA

The program FASTA [34] (with parameter settings: word size 2, gap opening penalty 10, gap extension penalty 10, no statistics, number of alignments up to 30000) was used to search for similarities between sequences of deregulated mRNAs and the siRNA (*GFP *or shuffled). This search is equivalent to the FASTA alignment of the siRNA or miRNA duplexes with the off-target transcripts used in [13]. All alignments were filtered for complete identical oligomers of 8 and more bases, 9 and more bases, etc. Additionally, the positions of the identical oligomers were extracted. Using the "cds" field in the annotation of the Refseq sequences, the region of the 3' UTR of the genes was defined and determined, whether the homologous region was located in the 3'UTR. As control genes, we used 207 mRNAs whose expression was not changed in the experiment and looked for sequence homologies in the same way (see Additional file 3). To have an appropriate control for the siRNA we used SHUFFLE (Wisconsin Package, Accelrys Inc.) to randomize the order of the nucleotides in the siRNAs without changing the composition (Figure 4). In an additional control, the sequences were randomized so that not only composition, but also dinucleotide content was preserved (see Additional file 3). All shuffled sequences were then compared with the list of deregulated and control genes. Different sets of non deregulated genes were used as negative controls ("not dereg.A"; "not dereg.B"; "diff.array") that had the same size of 207 genes as the set of actually down regulated genes after transfection of *GFP *siRNA ("dereg."). The first two control sets contain genes that were not deregulated after transfection of *GFP *siRNA in all 8 hybridizations (M<0.2; "not dereg.A and B"). The third set of genes ("diff.array") were not deregulated (M<0.2) in an additional unrelated microarray experiment. Error bars depict standard deviations between sense and antisense sequences (see Additional file 3).

To compare the sequence of sense and antisense *GFP *siRNA molecules with the sequences of the target genes *CYLD *(NM_015247) and *SOAT *(NM_003101), the software package SIMILARITY [35] implemented in W2H [36] was used to find local similarities between the siRNA sequences and the genes according to Jackson *et al*., [13].

## Authors' contributions

CT carried out experiments, participated in the design of the study and drafted the manuscript, AS performed experiments, AH–W performed the statistical analyses, WW and AB generated the bioinformatics data, LSB contributed to experiments, AP participated in the design of the study and helped to draft the manuscript, PL and DM conceived the study, participated in its design and helped to draft the manuscript.

All authors read and approved the final manuscript.

## Supplementary Material

Additional file 1 **Additional genes down regulated by the GFP siRNA as measured by Real-Time PCR**. Besides *CYLD *and *SOAT*, we measured by Real-Time PCR the mRNA levels of the off-target genes *RAB21*, *NUDT3 *and *TCF4 *that we identified in our microarray screen. Down modulation of the mRNA levels of these genes after transfection of *GFP *siRNA could be reproduced as for *CYLD *and *SOAT*. Shown are levels of off-target gene mRNA normalized to a mock-transfected control and to two housekeeping genes (see Methods). Click here for file

Additional file 2 Genes deregulated after transfection with *GFP *siRNA. Click here for file

Additional file 3 **mRNAs deregulated by GFP siRNA contain short sequences perfectly matched to the sense or antisense strand of GFP siRNA**. In order to define the background level of homologies to short sequence motifs, we i) shuffled the *GFP *siRNA sequence and ii) searched for homologies in genes that were either not deregulated or randomly picked. Shuffling of the *GFP *siRNA sequence was performed either with preserving the dinucleotide composition or without preservation of the dinucleotide composition (see Methods for details). The resulting shuffled *GFP *siRNA sequence was aligned to the set of genes that were deregulated by the proper *GFP *siRNA sequence in the profiling experiment ("shuffled dinucleotides preserved" and "shuffled dinucleotides not preserved"). For the negative control set of non-deregulated genes, we selected 207 genes that were least deregulated after transfection of *GFP *siRNA ("not dereg.A" and "not dereg. B") and 207 genes that were least deregulated in an unrelated profiling experiment ("diff.array", for details see Methods). We scored motifs of different lengths (ranging from >7 bp to >10 bp) that were homologous to the sequence of the transfected *GFP *siRNA. When comparing the sequence of *GFP *siRNA to sequences of the 207 significantly down regulated genes (grey bar), more genes with homology were found than in two sets of genes that were not regulated ("not dereg.A"; "not dereg.B") or taken from a non-related array ("diff.array"). With increasing length of homology, relatively more hits were scored for the proper, non-shuffled *GFP *siRNA in the set of actually deregulated genes as compared to all negative controls (bottom panels). Click here for file

Additional file 5 Description of normalization of microarray data. Click here for file

Additional file 4 qPCR primer sequences. Click here for file
