# Supplementary figures and images for: Off-target effects of siRNA specific for GFP
Source: BMC Mol Biol. 2008 Jun 24;9:60. doi: 10.1186/1471-2199-9-60 (PMC2443166; doi:10.1186/1471-2199-9-60)

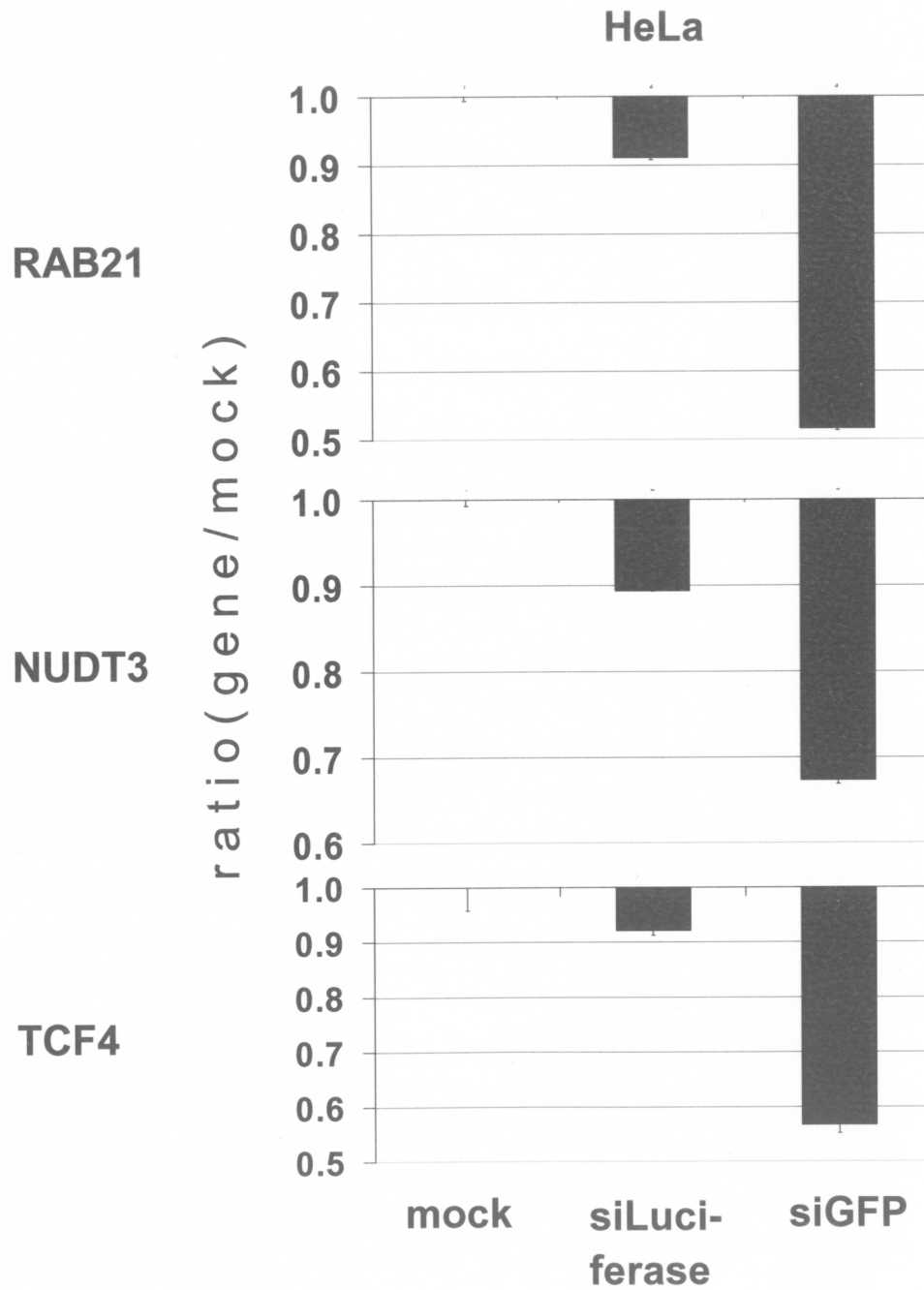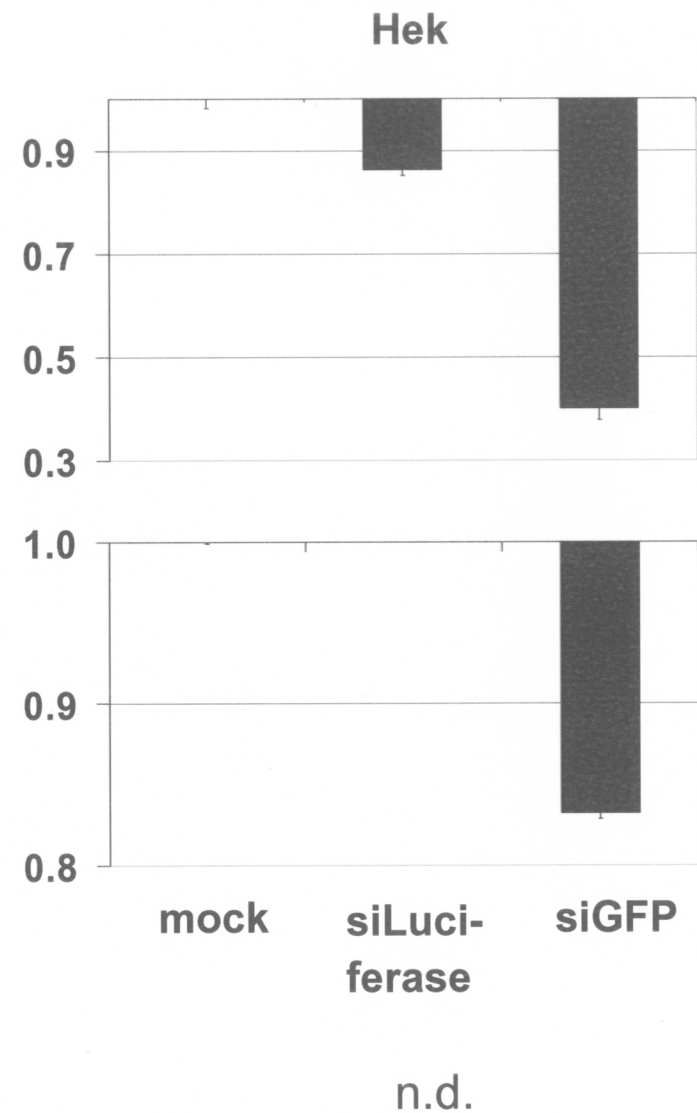

Supplement: Additional file 1 — Additional genes down regulated by the GFP siRNA as measured by Real-Time PCR. Besides CYLD and SOAT, we measured by Real-Time PCR the mRNA levels of the off-target genes RAB21, NUDT3 and TCF4 that we identified in our microarray screen. Down modulation of the mRNA levels of these genes after transfection of GFP siRNA could be reproduced as for CYLD and SOAT. Shown are levels of off-target gene mRNA normalized to a mock-transfected control and to two housekeeping genes (see Methods). [file 1471-2199-9-60-S1.pdf]
