# Supplementary material for: Off-target effects of siRNA specific for GFP
Source: BMC Mol Biol. 2008 Jun 24;9:60. doi: 10.1186/1471-2199-9-60 (PMC2443166; doi:10.1186/1471-2199-9-60)
Supplement: Additional file 2 — Genes deregulated after transfection with GFP siRNA. [file 1471-2199-9-60-S2.pdf]

Additional file 2: Genes deregulated after transfection with *GFP* siRNA

upregulated genes				HELA			HEK		
**#**	**name**	**RefSeq**	**molecular function**	**M**	**adj.P.Val**	**B**	**M**	**adj.P.Val**	**B**
1	ANXA3	NM_005139	diphosphoinositol-polyphosphate diphosphatase	2.75	1.13E-15	37.54	0.47	3.89E-05	9.08
2	LOC51149	NM_016175	hypothetical protein	1.29	3.48E-11	24.46	0.29	8.79E-03	1.61
3	DDAH1	NM_012137	hydrolase activity	1.02	1.87E-10	22.07	0.35	5.88E-04	5.27
4	UBP18	NM_017414.2	ubiquitin thiolesterase activity	1.00	1.99E-10	21.99	0.54	4.64E-06	11.91
5	IL15RA	NM_002189	interleukin receptor activity	1.01	2.06E-10	21.94	0.30	2.38E-03	3.34
6	SPHK1	NM_021972	calmodulin binding;transferase activity	1.01	2.16E-10	21.88	0.42	9.44E-05	7.85
7	HIST1H2AC	NM_003512.3	DNA binding	1.27	2.49E-10	21.68	0.54	7.94E-05	8.08
8	MAG1	NM_032717	acyltransferase activity	0.95	2.70E-10	21.53	0.40	1.21E-04	7.53
9	SAT1	NM_002970	acyltransferase activity	1.08	3.21E-10	21.24	0.45	1.46E-04	7.25
10	PTGS2	NM_000963	peroxidase activity;oxidoreductase activity	1.25	4.65E-10	20.78	0.38	3.44E-03	2.84
11	HDHD1A	NM_012080	catalytic activity	0.95	8.31E-10	20.03	0.35	8.84E-04	4.67
12	STAMBPL1	NM_020799	ubiquitin thiolesterase activity	0.80	9.32E-10	19.90	0.30	8.23E-04	4.78
13	PIGL	NM_004278	hydrolase activity	0.83	1.17E-09	19.61	0.36	2.10E-04	6.73
14	CLPB	NM_030813	nucleoside-triphosphatase activity	0.81	2.28E-09	18.74	0.28	3.67E-03	2.75
15	SMPDL3B	NM_014474	hydrolase activity, acting on glycosyl bonds	0.88	2.40E-09	18.67	0.33	1.45E-03	4.02
16	HERC6	NM_017912	ubiquitin-protein ligase activity;GEF activity	0.81	2.69E-09	18.52	0.43	5.32E-05	8.62
17	GLRB	NM_000824	inhibitory glycine receptor, CNS	0.79	4.31E-09	17.90	0.28	3.47E-03	2.82
18	HHLA3	NM_007071	unknown	0.73	5.02E-09	17.71	0.36	1.86E-04	6.89
19	GNPDA1	NM_005471	hydrolase activity	0.63	2.46E-08	15.68	0.36	1.54E-04	7.17
20	PLEKHA7	NM_175058	unknown	0.63	3.29E-08	15.32	0.28	1.97E-03	3.59
21	RRP9	NM_004704	RNA binding	0.66	3.35E-08	15.30	0.28	3.01E-03	3.02
22	POLR3H	NM_138338	protein binding;transferase activity	0.70	5.10E-08	14.79	0.34	1.41E-03	4.05
23	PLSCR4	NM_020353	calcium ion binding	0.70	5.50E-08	14.70	0.30	4.35E-03	2.53
24	PSEN2	NM_012486	protein binding;peptidase activity	0.61	5.87E-08	14.62	0.26	4.64E-03	2.45
25	URG4	NM_017920	GTP binding	0.62	6.96E-08	14.39	0.36	2.91E-04	6.26
26	USP18	NM_017414	ubiquitin thiolesterase activity	0.80	7.32E-08	14.32	0.56	4.38E-05	8.92
27	LRRC1	NM_018214	transferase activity	0.65	8.39E-08	14.15	0.26	9.21E-03	1.55
28	TPBG	NM_006670	protein binding	0.79	8.93E-08	14.07	0.40	1.33E-03	4.14
29	IFIT3	NM_001549	binding	0.84	1.07E-07	13.84	0.46	7.07E-04	4.99
30	LMO7	NM_005358	actin binding	0.59	1.07E-07	13.83	0.38	1.50E-04	7.21
31	~	XR_017744.1	unknown	0.65	1.09E-07	13.81	0.36	5.93E-04	5.26
32	IFIH1	NM_022168	RNA binding;helicase activity	0.64	1.82E-07	13.16	0.28	9.11E-03	1.56
33	SNX10	NM_013322	protein binding;phosphoinositide binding	0.62	2.29E-07	12.88	0.41	1.70E-04	7.03
34	HIST1H1C	NM_005319	DNA binding;protein binding	0.82	2.94E-07	12.58	0.49	6.71E-04	5.06
35	BARD1	NM_000465	RNA binding;ubiquitin-protein ligase activity	0.52	3.84E-07	12.25	0.24	6.93E-03	1.92
36	VCL	NM_014000	structural molecule;oxidoreductase activity	0.57	3.93E-07	12.22	0.29	3.59E-03	2.78
37	TRIOBP	NM_138632	TRIO and F-actin binding protein	0.53	3.99E-07	12.20	0.25	6.42E-03	2.01
38	ALG6	NM_013339	transferase activity	0.54	4.71E-07	11.99	0.39	1.28E-04	7.45
39	FNDC3A	NM_014923	unknown	0.57	4.98E-07	11.92	0.41	1.33E-04	7.38
40	REEP1	NM_022912	olfactory receptor binding	0.51	6.17E-07	11.67	0.61	5.88E-07	14.69
41	C17orf68	NM_025099	unknown	0.68	6.95E-07	11.52	0.44	5.35E-04	5.41
42	ABHD3	NM_138340	serine esterase activity;hydrolase activity	0.53	7.48E-07	11.43	0.32	8.79E-04	4.68
43	NMI	NM_004688	transcription cofactor activity;protein binding	0.54	7.75E-07	11.38	0.45	4.56E-05	8.85
44	SCRT2	NM_080725	antioxidant activity;oxidoreductase activity	0.55	8.47E-07	11.27	0.38	2.91E-04	6.26
45	MLL3	NM_170606	transferase activity	0.53	8.47E-07	11.27	0.34	6.61E-04	5.09
46	PSMD4	NM_002810	protein binding	0.60	8.52E-07	11.26	0.30	6.59E-03	1.98
47	SEC22A	NM_012430	transporter activity	0.51	9.77E-07	11.09	0.29	2.40E-03	3.32
48	DOLK	NM_014908	phosphatidate cytidylyltransferase activity	0.49	1.00E-06	11.05	0.24	7.98E-03	1.73
49	MYL6	NM_021019	actin-dependent ATPase activity	0.59	1.12E-06	10.91	0.32	4.02E-03	2.63
50	ANXA1	NM_000700	phospholipase inhibitor activity;receptor binding	0.53	1.33E-06	10.71	0.35	6.92E-04	5.02
51	RIBC2	NM_015653	unknown	0.48	1.47E-06	10.58	0.35	3.16E-04	6.13
52	LGALS3BP	NM_005567	scavenger receptor activity;protein binding	0.47	1.48E-06	10.58	0.24	8.11E-03	1.71
53	TNR6C	NM_018996.2	nucleotide binding	0.52	1.56E-06	10.51	0.26	8.93E-03	1.59
54	TFRC	NM_003234	receptor activity;transferrin receptor activity	0.49	1.60E-06	10.48	0.36	3.42E-04	6.01
55	THAS	NM_001061.2	electron transport	0.62	1.64E-06	10.45	0.32	8.90E-03	1.59
56	MCOLN3	NM_018298	ion channel activity	0.52	1.70E-06	10.40	0.35	8.29E-04	4.76
57	CBR4	NM_032783	oxidoreductase activity	0.51	1.71E-06	10.40	0.32	1.54E-03	3.94
58	TESC	NM_017899	calcium ion binding	0.56	1.76E-06	10.35	0.50	4.52E-05	8.87
59	FBN1	NM_000138	extracellular matrix structural constituent	0.62	1.76E-06	10.36	0.31	1.04E-02	1.40
60	CASP7	NM_001227	cysteine-type peptidase activity;caspase activity	0.52	1.86E-06	10.29	0.40	1.76E-04	6.96
61	MAPKAPK3	NM_004635	protein serine/threonine kinase activity	0.52	2.14E-06	10.11	0.40	2.64E-04	6.41
62	CRY1	NM_004075	G-protein coupled photoreceptor activity	0.56	2.30E-06	10.02	0.44	1.81E-04	6.92
63	GBA	NM_000157	hydrolase activity	0.58	2.31E-06	10.01	0.30	1.04E-02	1.40
64	HN1	NM_001002032	unknown	0.56	2.41E-06	9.95	0.49	6.49E-05	8.37
65	LIMA1	NM_016357	actin monomer binding;actin filament binding	0.54	2.62E-06	9.85	0.33	2.48E-03	3.28
66	C13orf27	NM_138779	unknown	0.59	3.03E-06	9.66	0.36	2.75E-03	3.14
67	HSD17B6	NM_003725	oxidoreductase activity	0.50	3.63E-06	9.43	0.38	3.50E-04	5.97
68	SLC44A3	NM_152369	unknown	0.54	3.64E-06	9.43	0.51	4.12E-05	9.01
69	SERPINI1	NM_005025	serine-type endopeptidase inhibitor activity	0.47	3.89E-06	9.35	0.46	3.75E-05	9.13
70	LOC497661	NM_001035005.2	unknown	0.53	4.19E-06	9.25	0.42	3.10E-04	6.16

71	DUS2L	NM_017803	double-stranded RNA binding	0.49	4.20E-06	9.25	0.32	1.85E-03	3.69
72	TXNRD1	NM_182743	oxidoreductase activity	0.46	4.58E-06	9.14	0.30	2.13E-03	3.48
73	TRXR1	NM_182742	signal transduction	0.46	4.58E-06	9.14	0.30	2.13E-03	3.48
74	TPD52L1	NM_003287	protein dimerization activity	0.48	6.54E-06	8.69	0.28	8.47E-03	1.66
75	REEP3	NM_001001330.1	integral to membrane	0.52	7.13E-06	8.59	0.34	3.37E-03	2.86
76	PIP5K2C	NM_024779	kinase activity;transferase activity	0.46	7.26E-06	8.57	0.37	4.36E-04	5.68
77	EWS	NM_005243.2	regulation of transcription	0.45	7.54E-06	8.52	0.27	6.46E-03	2.01
78	EFEMP1	NM_004105	calcium ion binding;protein binding	0.48	8.44E-06	8.38	0.32	2.48E-03	3.28
79	FBLN3	NM_004105	unknown	0.48	8.44E-06	8.38	0.32	2.48E-03	3.28
80	FZD3	NM_017412	G-protein coupled receptor activity	0.42	9.55E-06	8.23	0.32	7.81E-04	4.84
81	TRIM45	NM_025188	protein binding;zinc ion binding	0.44	9.67E-06	8.22	0.38	2.87E-04	6.30
82	FLJ38973	NM_153689	hypothetical protein	0.47	9.91E-06	8.19	0.45	7.15E-05	8.23
83	TFPI	NM_006287	serine-type endopeptidase inhibitor activity	0.51	1.01E-05	8.16	0.35	1.96E-03	3.59
84	MAPE	NM_006115.3	unknown	0.45	1.03E-05	8.13	0.45	5.15E-05	8.67
85	HOXA1	NM_005522	RNA polymerase II transcription factor activity	0.43	1.04E-05	8.13	0.44	4.57E-05	8.84
86	ARSJ	NM_024590	hydrolase activity	0.44	1.06E-05	8.10	0.37	3.42E-04	6.00
87	SLC16A10	NM_018593	transporter activity	0.46	1.29E-05	7.87	0.30	5.04E-03	2.33
88	FBXO4	NM_012176	ubiquitin-protein ligase activity;protein binding	0.44	1.30E-05	7.87	0.29	3.19E-03	2.94
89	MAFB	NM_005461	DNA binding;transcription factor activity	0.40	1.34E-05	7.83	0.35	3.15E-04	6.14
90	NIT1	NM_005600	hydrolase activity	0.43	1.47E-05	7.71	0.31	1.74E-03	3.77
91	NRG4	NM_138573	growth factor activity	0.40	1.55E-05	7.65	0.35	2.90E-04	6.27
92	MARS	NM_004990	methionine-tRNA ligase activity	0.47	1.61E-05	7.60	0.42	3.14E-04	6.14
93	ZMYM6	NM_007167.3	protein binding	0.44	1.72E-05	7.52	0.28	7.23E-03	1.86
94	ELOVL1	NM_022821	protein binding	0.43	1.76E-05	7.48	0.65	9.37E-07	14.02
95	SLC1A3	NM_004172	L-glutamate transporter activity	0.45	1.80E-05	7.46	0.46	7.45E-05	8.16
96	KIAA0746	NM_015187	binding	0.48	1.95E-05	7.36	0.39	7.81E-04	4.85
97	B4GALT6	NM_004775	transferase activity	0.39	2.15E-05	7.24	0.36	2.31E-04	6.59
98	TIPARP	NM_015508	protein binding;transferase activity	0.45	2.38E-05	7.12	0.29	6.59E-03	1.98
99	Q86VR7	XM_085831	unknown	0.44	2.38E-05	7.12	0.33	1.95E-03	3.61
100	HSPA4L	NM_014278	nucleotide binding;protein binding;ATP binding	0.40	2.66E-05	6.99	0.25	8.78E-03	1.61
101	NSF	NM_006178	ATP binding;hydrolase activity	0.43	2.67E-05	6.98	0.44	9.72E-05	7.81
102	ZDHHC23	NM_173570	acyltransferase activity	0.45	2.70E-05	6.97	0.62	3.63E-06	12.22
103	STAR9	XM_001129290.1	unknown	0.37	3.51E-05	6.66	0.24	9.94E-03	1.45
104	FGG	NM_021870	receptor binding;calcium ion binding	0.42	3.89E-05	6.53	0.29	5.62E-03	2.18
105	GLMN	NM_053274	hepatocyte growth factor receptor binding	0.36	4.14E-05	6.46	0.42	3.75E-05	9.13
106	CREBL2	NM_001310	transcription factor activity	0.38	4.28E-05	6.41	0.30	1.83E-03	3.70
107	CCNC	NM_005190	transcriptional regulation	0.50	4.36E-05	6.39	0.35	5.37E-03	2.25
108	DNAJC5	NM_025219.1	protein folding	0.41	4.58E-05	6.33	0.35	9.72E-04	4.55
109	STX12	NM_177424	protein transporter activity	0.40	5.00E-05	6.23	0.33	1.38E-03	4.09
110	TMEM55A	NM_018710	hydrolase activity	0.44	5.10E-05	6.20	0.31	5.38E-03	2.25
111	PLAGL2	NM_002657	transcription factor activity;zinc ion binding	0.37	5.14E-05	6.19	0.34	4.94E-04	5.51
112	PIGY	NM_032906	protein binding	0.38	5.70E-05	6.12	0.36	2.12E-03	3.65
113	CHAC2	NM_001008708	cation transport activity	0.36	6.07E-05	5.98	0.45	2.46E-05	9.72
114	SNX11	NM_152244	protein binding;phosphoinositide binding	0.39	6.40E-05	5.92	0.29	4.92E-03	2.37
115	ACTR5	NM_024855	protein binding;ATP binding	0.40	8.14E-05	5.63	0.45	9.91E-05	7.78
116	NSMAF	NM_003580	receptor signaling protein activity	0.37	8.43E-05	5.59	0.32	1.35E-03	4.12
117	TMEM9B	NM_020644	signal transducer activity	0.37	8.52E-05	5.58	0.37	3.93E-04	5.82
118	INPP5A	NM_005539	phosphatidylinositol phosphatase activity	0.40	8.55E-05	5.57	0.36	9.46E-04	4.59
119	PDCD10	NM_007217	protein binding	0.43	9.57E-05	5.44	0.41	5.37E-04	5.40
120	GNB5	NM_016194	GTPase activity;signal transducer activity	0.35	1.13E-04	5.25	0.25	9.11E-03	1.56
121	CR2	NM_001877	complement receptor activity	0.37	1.19E-04	5.18	0.45	5.43E-05	8.60
122	SLIT2	NM_004787	follicle-stimulating hormone receptor activity	0.36	1.30E-04	5.09	0.27	6.59E-03	1.98
123	SS18L2	NM_016305	unknown	0.35	1.44E-04	4.96	0.25	9.10E-03	1.56
124	C19orf63	NM_175063	unknown	0.36	1.55E-04	4.87	0.43	8.41E-05	7.99
125	ABCF2	NM_007189	transporter activity;ATPase activity	0.38	1.61E-04	4.82	0.32	2.28E-03	3.39
126	PHLPPL	NM_015020	phosphoprotein phosphatase activity	0.33	1.86E-04	4.65	0.28	3.10E-03	2.98
127	GPR160	NM_014373	rhodopsin-like receptor activity	0.34	1.95E-04	4.59	0.35	5.75E-04	5.30
128	SLC27A2	NM_003645	long-chain-fatty-acid-CoA ligase activity	0.37	2.04E-04	4.54	0.40	2.89E-04	6.28
129	LYRM5	NM_001001660	unknown	0.35	2.08E-04	4.52	0.34	8.16E-04	4.79
130	GCN1L1	NM_006836	translation factor activity	0.41	2.19E-04	4.46	0.32	6.30E-03	2.04
131	PKIA	NM_006823	cAMP-dependent protein kinase inhibitor activity	0.38	2.26E-04	4.42	0.41	4.07E-04	5.78
132	PFN2	NM_053024	actin binding;protein binding	0.36	2.28E-04	4.41	0.54	1.34E-05	10.54
133	PSMC3	NM_002804	transcription regulation activity	0.37	2.37E-04	4.36	0.30	5.91E-03	2.12
134	MOSC2	NM_017898	oxidoreductase activity	0.36	2.46E-04	4.31	0.32	2.12E-03	3.49
135	LIMCH1	NM_014988	actin binding;zinc ion binding	0.36	2.70E-04	4.20	0.31	3.78E-03	2.71
136	OAS3	NM_006187	RNA binding;ATP binding;transferase activity	0.40	2.82E-04	4.15	0.80	5.77E-07	14.75
137	PLS1	NM_002670.1	actin binding	0.39	2.94E-04	4.10	0.48	1.34E-04	7.37
138	SLC35A5	NM_017945	nucleotide-sugar transporter activity	0.32	3.26E-04	3.98	0.38	2.19E-04	6.67
139	MPHOSPH6	NM_005792	protein binding	0.38	3.35E-04	3.94	0.40	6.49E-04	5.12
140	PARP4	NM_006437	NAD+ ADP-ribosyltransferase activity	0.35	3.69E-04	3.83	0.33	2.08E-03	3.51
141	ZWINT	NM_007057.3	mitotic check point	0.30	3.75E-04	3.81	0.53	3.21E-06	12.40
142	SDC2	NM_002998	molecular_function;cytoskeletal protein binding	0.35	3.86E-04	3.78	0.35	1.31E-03	4.16
143	VPS16	NM_002836	receptor activity	0.34	3.87E-04	3.78	0.27	8.40E-03	1.66

144	PTPRA	NM_002836	receptor activity	0.34	3.87E-04	3.78	0.27	8.40E-03	1.66
145	ARL2BP	NM_012106	small GTPase regulator activity	0.34	4.49E-04	3.59	0.41	2.74E-04	6.36
146	NUB1	NM_016118	molecular_function	0.30	5.45E-04	3.37	0.38	1.48E-04	7.22
147	PTER	NM_030664	zinc ion binding;hydrolase activity	0.30	5.48E-04	3.36	0.24	9.37E-03	1.52
148	PLEKHH2	NM_172069	binding	0.41	5.56E-04	3.34	0.36	5.54E-03	2.21
149	PISD	NM_014338	phosphatidylserine decarboxylase activity	0.31	5.77E-04	3.30	0.48	1.96E-05	10.06
150	EML4	NM_019063	molecular_function;protein binding	0.35	5.77E-04	3.30	0.47	1.09E-04	7.66
151	RABGAP1	NM_012197	tubulin binding;regulation of Rab GTPase activity	0.30	5.90E-04	3.27	0.31	1.47E-03	4.00
152	SPINT2	NM_021102	serine-type endopeptidase inhibitor activity	0.34	5.95E-04	3.26	0.28	9.43E-03	1.52
153	SLC20A1	NM_005415	receptor activity	0.39	6.61E-04	3.13	0.39	1.98E-03	3.58
154	SCOC	NM_032547	unknown	0.33	7.28E-04	3.02	0.37	7.51E-04	4.91
155	CD59	NM_203331	protein binding;GPI anchor binding	0.34	7.34E-04	3.01	0.30	6.13E-03	2.07
156	RTN4IP1	NM_032730	zinc ion binding;oxidoreductase activity	0.30	7.72E-04	2.95	0.46	3.15E-05	9.36
157	HIP2	NM_005339	ubiquitin-protein ligase activity	0.33	7.81E-04	2.94	0.31	3.57E-03	2.79
158	C3orf59	NM_178496.2	unknown	0.31	7.87E-04	2.93	0.37	3.16E-04	6.12
159	C1orf56	NM_006818	unknown	0.29	8.11E-04	2.89	0.27	5.42E-03	2.23
160	MLLT11	NM_006818	unknown	0.29	8.11E-04	2.89	0.27	5.42E-03	2.23
161	HLA-DRB5	NM_002125	antigen presentation via MHC class II	0.28	8.69E-04	2.81	0.28	2.92E-03	3.06
162	STK11IP	NM_052902	protein binding;kinase activity	0.28	8.76E-04	2.80	0.27	3.54E-03	2.80
163	GRAMD1C	NM_017577.3	unknown	0.34	8.94E-04	2.77	0.46	1.72E-04	7.01
164	CKLF	NM_016951	chemokine activity	0.30	9.13E-04	2.75	0.28	4.88E-03	2.38
165	PCOLCE2	NM_013363	heparin binding	0.32	9.16E-04	2.75	0.37	6.64E-04	5.08
166	SLC25A12	NM_003705	L-glutamate transporter activity	0.31	9.81E-04	2.67	0.28	5.96E-03	2.11
167	COIL	NM_004645	protein binding	0.30	9.82E-04	2.66	0.27	5.55E-03	2.20
168	PIK3CB	NM_006219	insulin receptor substrate binding;kinase activity	0.30	1.00E-03	2.64	0.27	7.22E-03	1.87
169	PKIB	NM_181795	cAMP-dependent protein kinase inhibitor activity	0.29	1.03E-03	2.61	0.42	6.72E-05	8.32
170	SLC41A1	NM_173854	cation transporter activity	0.30	1.03E-03	2.60	0.41	1.67E-04	7.06
171	DMD	NM_000109	actin binding;structural constituent of cytoskeleton	0.27	1.06E-03	2.57	0.37	1.71E-04	7.02
172	CD58	NM_001779	protein binding;GPI anchor binding	0.28	1.17E-03	2.46	0.26	6.80E-03	1.94
173	PRPSAP2	NM_002767	enzyme inhibitor activity	0.30	1.22E-03	2.41	0.27	1.00E-02	1.44
174	PSMC5	NM_002805	transcription cofactor activity	0.29	1.26E-03	2.37	0.33	1.16E-03	4.32
175	ATP5L	NM_006476	hydrogen ion transporting ATPase activity	0.27	1.31E-03	2.32	0.34	4.26E-04	5.72
176	CEP72	NM_018140	protein binding	0.33	1.34E-03	2.30	0.29	1.03E-02	1.40
177	FMO5	NM_001461	monooxygenase activity;FAD binding	0.28	1.34E-03	2.30	0.27	4.69E-03	2.43
178	TFDP1	NM_007111	transcription factor activity	0.31	1.41E-03	2.24	0.52	2.37E-05	9.81
179	GTF2A2	NM_004492	RNA polymerase II transcription factor activity	0.27	1.43E-03	2.22	0.30	1.49E-03	3.98
180	ARHGEF4	NM_015320	Rho guanyl-nucleotide exchange factor activity	0.29	1.46E-03	2.20	0.33	1.10E-03	4.38
181	EBNA1BP2	NM_006824	protein binding	0.31	1.57E-03	2.11	0.41	2.64E-04	6.40
182	MTMR9	NM_015458	phosphatidylinositol phosphatase activity	0.33	1.67E-03	2.04	0.33	5.12E-03	2.31
183	FARS2	NM_006567	phenylalanine-tRNA ligase activity	0.33	1.92E-03	1.87	0.34	3.16E-03	2.95
184	SYNE1	NM_182961	actin binding;structural molecule activity	0.29	2.00E-03	1.82	0.27	7.78E-03	1.76
185	CTSC	NM_148170	cysteine-type peptidase activity	0.27	2.12E-03	1.76	0.44	4.99E-05	8.71
186	DYNC1LI1	NM_016141	nucleotide binding;motor activity;ATP binding	0.27	2.13E-03	1.75	0.27	5.57E-03	2.20
187	HEL308	NM_133636	ATP-dependent helicase activity	0.26	2.26E-03	1.68	0.29	1.73E-03	3.77
188	IGFBP7	NM_001553	insulin-like growth factor binding	0.27	2.47E-03	1.57	0.31	1.53E-03	3.94
189	ALS2	NM_020919	guanyl-nucleotide exchange factor activity	0.27	2.57E-03	1.53	0.26	7.57E-03	1.80
190	FAM49B	NM_016623	unknown	0.27	2.82E-03	1.42	0.26	9.03E-03	1.57

Additional file 2: Genes deregulated after transfection with *GFP* siRNA

downregulated genes				HELA			HEK		
**#**	**name**	**RefSeq/Ensembl**	**molecular function**	**M**	**adj.P.Val**	**B**	**M**	**adj.P.Val**	**B**
1	SOAT1	NM_003101	sterol O-acyltransferase activity	-2.28	8.72E-14	32.76	-0.83	8.18E-07	14.15
2	Q96HQ8	ENSG00000173215	unknown	-1.95	8.72E-14	32.87	-1.52	7.71E-12	27.43
3	CASP8	NM_033356	signal transducer activity;caspase activity	-1.56	5.35E-13	30.32	-0.88	6.42E-09	20.37
4	C10orf56	NM_153367	nucleic acid binding;zinc ion binding	-1.62	6.03E-13	30.12	-0.49	1.35E-05	10.51
5	NUDT3	NM_006703	magnesium ion binding;hydrolase activity	-1.45	1.74E-12	28.78	-0.42	6.64E-05	8.35
6	RAB21	NM_014999	nucleotide binding;GTP binding	-1.55	1.89E-12	28.60	-0.76	1.51E-07	16.52
7	HMGCS1	NM_002130	hydroxymethylglutaryl-CoA synthase activity	-1.48	2.73E-12	28.09	-0.45	6.05E-05	8.47
8	PAICS	NM_006452	catalytic activity;ATP binding	-1.37	3.72E-12	27.62	-0.66	3.61E-07	15.39
9	TM16F	NM_001025356.1	integral to membrane	-1.59	4.96E-12	27.25	-0.45	2.18E-04	6.68
10	GOSR1	NM_004871	v-SNARE activity	-1.47	5.13E-12	27.13	-0.46	6.89E-05	8.29
11	PSF1	NM_021067.3	protein binding	-2.18	5.44E-12	27.01	-1.55	5.47E-09	20.81
12	CYLD	NM_015247	NFkB pathway	-1.39	6.75E-12	26.74	-0.60	2.91E-06	12.56
13	CXorf34	NM_024917	methyltransferase activity	-1.68	1.11E-11	26.08	-0.67	1.08E-05	10.82
14	SKP2	NM_005983	protein binding	-1.18	1.80E-11	25.33	-0.74	7.49E-08	17.28
15	IL6ST	NM_002184	interleukin-6 receptor activity;protein binding	-1.20	2.92E-11	24.72	-0.57	3.49E-06	12.27
16	C10orf119	NM_024834	hypothetical protein	-1.14	3.13E-11	24.63	-0.26	5.18E-03	2.29
17	LARS2	NM_015340	leucine-tRNA ligase activity;ATP binding	-1.18	5.29E-11	23.85	-0.49	2.65E-05	9.59
18	NR1D2	NM_005126	transcription factor activity	-1.12	5.63E-11	23.71	-0.32	1.36E-03	4.11
19	ACSL4	NM_022977	long-chain-fatty-acid-CoA ligase activity	-1.22	6.44E-11	23.51	-0.47	7.21E-05	8.21
20	CCD25	NP_060716	unknown	-1.09	1.72E-10	22.18	-0.33	1.67E-03	3.83
21	RPIA	NM_144563	ribose-5-phosphate isomerase activity	-1.25	2.70E-10	21.55	-0.36	4.29E-03	2.55
22	ST3GAL4	NM_006278	ST3 beta-galactoside sialyltransferase 4	-0.88	2.70E-10	21.49	-0.29	1.31E-03	4.16
23	Q8NDF8-3	NM_001040284	unknown	-0.88	2.92E-10	21.37	-0.37	1.30E-04	7.41
24	TCF4	NM_003199	transcription factor activity	-1.00	3.51E-10	21.12	-0.38	3.16E-04	6.12
25	UNC5B	NM_170744	signal transduction	-1.01	4.59E-10	20.80	-0.33	1.95E-03	3.61
26	MMP2	NM_004530	protein binding, metal ion binding	-0.99	5.87E-10	20.49	-0.27	9.65E-03	1.48
27	ETS2	NM_005239	transcription factor activity	-0.82	9.78E-10	19.83	-0.38	9.55E-05	7.84
28	YTHDF1	NM_017798	unknown	-0.80	1.03E-09	19.76	-0.58	7.44E-07	14.38
29	HPRT1	NM_000194	hypoxanthine phosphoribosyltransferase	-0.99	1.58E-09	19.23	-1.58	3.06E-11	26.02
30	HP1BP3	NM_016287	DNA binding	-0.78	2.89E-09	18.43	-0.40	9.62E-05	7.82
31	ZFAND6	NM_019006	DNA binding;zinc ion binding	-0.97	3.18E-09	18.31	-0.33	4.55E-03	2.47
32	RAB4A	NM_004578	GTPase activity	-0.94	3.23E-09	18.28	-0.30	8.50E-03	1.65
33	MAPK9	NM_002752	protein serine/threonine kinase activity	-0.89	4.63E-09	17.82	-0.59	7.67E-06	11.24
34	CYFIP1	NM_014608	Rac GTPase binding;actin filament binding	-0.75	5.30E-09	17.63	-0.51	6.86E-06	11.36
35	ZNF503	NM_032772	nucleic acid binding;zinc ion binding	-1.06	6.64E-09	17.35	-0.56	1.29E-04	7.43
36	~	ENSG00000187025	unknown	-1.08	6.78E-09	17.33	-0.39	4.92E-03	2.37
37	SUHW3	NM_017666	DNA binding;zinc ion binding	-0.80	7.21E-09	17.24	-0.28	5.66E-03	2.18
38	GAL	NM_015973	neuropeptide hormone activity	-1.29	1.04E-08	16.76	-0.71	1.11E-04	7.64
39	EEF1A2	NM_001958	translation elongation factor activity	-0.69	1.91E-08	16.01	-0.33	6.60E-04	5.10
40	ERRFI1	NM_018948	Rho GTPase activator activity	-0.73	2.28E-08	15.78	-0.32	1.70E-03	3.80
41	OAZ1	NM_004152	ornithine decarboxylase inhibitor activity	-0.75	2.32E-08	15.76	-0.40	2.92E-04	6.26
42	ALS2CR13	NM_173511	unknown	-0.68	2.37E-08	15.73	-0.30	1.79E-03	3.74
43	THBS2	NM_003247	structural molecule activity	-0.76	2.97E-08	15.44	-0.28	8.87E-03	1.60
44	C5orf5	NM_016603	GTPase activator activity	-0.68	3.04E-08	15.41	-0.33	1.03E-03	4.48
45	FLJ31818	NM_152556	unknown	-0.68	3.27E-08	15.33	-0.27	5.38E-03	2.24
46	~	ENSG00000180632	unknown	-0.81	5.10E-08	14.79	-0.71	2.12E-06	12.90
47	LOC146909	NP_001073912	ATP binding	-0.66	5.40E-08	14.72	-0.95	5.47E-09	20.75
48	Q8N2D2	ENST00000356486	unknown	-0.65	6.12E-08	14.57	-0.33	1.01E-03	4.50
49	TRAK2	NM_015049	intracellular transporter activity	-0.63	6.65E-08	14.45	-0.29	2.35E-03	3.35
50	SERINC1	NM_020755	protein binding;L-serine transporter activity	-0.89	8.05E-08	14.20	-0.38	5.01E-03	2.34
51	HIPK1	NM_198268	protein serine/threonine kinase activity	-0.76	8.10E-08	14.19	-0.34	3.56E-03	2.79
52	C13orf24	NM_006346	unknown	-0.78	8.21E-08	14.18	-0.32	8.71E-03	1.62
53	TTC21A	NM_145755	binding	-0.69	8.21E-08	14.18	-0.43	1.84E-04	6.91
54	ERBB2IP	NM_018695	unknown	-0.59	1.07E-07	13.84	-0.42	4.78E-05	8.77
55	PDK1	NM_002610	protein histidine kinase activity	-0.71	1.15E-07	13.75	-0.35	1.88E-03	3.66
56	ZFX	NM_003410	transcription coactivator activity	-0.62	1.29E-07	13.59	-0.40	1.52E-04	7.19
57	CDON	NM_016952	protein binding	-0.58	1.40E-07	13.48	-0.26	5.14E-03	2.31
58	GYLTL1B	NM_152312	transferase activity	-0.59	1.86E-07	13.13	-0.63	7.22E-07	14.42
59	TSC22D1	NM_006022	transcription factor activity	-0.69	2.10E-07	12.98	-0.54	2.88E-05	9.49
60	C14orf172	NM_152307	unknown	-0.67	2.29E-07	12.88	-0.37	1.15E-03	4.32
61	VLDLR	NM_003383	receptor activity;lipid transporter activity	-0.69	2.40E-07	12.82	-0.56	2.08E-05	9.96
62	STX2	NM_194356	protein transporter activity	-0.65	3.40E-07	12.40	-0.44	2.05E-04	6.77
63	CCDC71	NM_022903	unknown	-0.66	3.46E-07	12.38	-0.42	3.42E-04	6.00
64	OCRL	NM_001587	phosphatidylinositol phosphatase activity	-0.59	4.10E-07	12.17	-0.57	4.64E-06	11.90
65	ZBTB44	NM_014155	DNA binding;protein binding;zinc ion binding	-0.61	4.70E-07	12.00	-0.58	6.62E-06	11.44
66	GAB1	NM_002039	signal transducer activity	-0.58	4.76E-07	11.97	-0.27	8.74E-03	1.62
67	SGTB	NM_019072	binding	-0.64	5.65E-07	11.77	-0.58	1.46E-05	10.42
68	ZNF587	NM_032828	DNA binding;zinc ion binding	-0.54	5.86E-07	11.73	-0.37	2.89E-04	6.28

69	PTPRU	NM_005704	protein tyrosine phosphatase activity	-0.87	6.22E-07	11.66	-0.53	8.05E-04	4.81
70	LMNB1	NM_005573	structural molecule activity	-0.61	7.58E-07	11.41	-0.36	1.57E-03	3.91
71	ARPP-19	NM_006628	potassium channel regulator activity	-0.53	1.05E-06	11.00	-0.63	9.50E-07	14.00
72	CE170	NM_014812	unknown	-0.50	1.07E-06	10.97	-0.40	1.05E-04	7.72
73	GHRHR	NM_000823	receptor activity	-0.56	1.18E-06	10.84	-0.49	3.51E-05	9.23
74	SS18L1	NM_198935	protein binding	-0.55	1.39E-06	10.65	-0.67	1.00E-06	13.90
75	TSTA3	NM_003313	electron carrier activity	-0.68	1.49E-06	10.57	-0.51	2.32E-04	6.58
76	ATAD5	NM_024857	nucleotide binding;ATP binding	-0.48	1.54E-06	10.53	-0.29	1.92E-03	3.63
77	SKP2	NM_005983	protein binding	-0.52	1.65E-06	10.44	-0.29	4.42E-03	2.51
78	STAG2	NM_006603	component of cohesin complex	-0.63	1.65E-06	10.44	-0.32	8.23E-03	1.69
79	TRIM65	NM_173547	protein binding;zinc ion binding	-0.54	1.72E-06	10.39	-0.52	2.08E-05	9.96
80	RGPD5	NM_005054	binding	-0.49	2.06E-06	10.16	-0.47	2.04E-05	9.99
81	RGPD7	NM_032260	binding	-0.49	2.06E-06	10.16	-0.47	2.04E-05	9.99
82	MOBK1B	NM_018221	protein binding;zinc ion binding	-0.50	2.44E-06	9.94	-0.38	3.42E-04	6.02
83	ZFP64	NM_199426	nucleic acid binding;zinc ion binding	-0.51	2.76E-06	9.78	-0.47	4.23E-05	8.96
84	C16orf63	NM_144600	unknown	-0.49	2.93E-06	9.71	-0.60	1.64E-06	13.25
85	STCH	NM_006948	nucleotide binding;ATP binding	-0.48	3.04E-06	9.65	-0.49	1.46E-05	10.42
86	SLC25A32	NM_030780	folic acid transporter activity	-0.45	3.70E-06	9.41	-0.38	1.32E-04	7.39
87	PLXB2	XM_371474	receptor activity	-0.49	3.84E-06	9.36	-0.52	1.34E-05	10.54
88	RREB1	NM_002955	nucleic acid binding;zinc ion binding	-0.48	3.89E-06	9.35	-0.50	1.48E-05	10.40
89	SH3GL1	NM_003025	protein binding	-0.71	4.07E-06	9.29	-0.38	9.52E-03	1.50
90	AKT1	NM_005163	protein serine/threonine kinase activity	-0.46	4.25E-06	9.23	-0.35	4.40E-04	5.67
91	ATP6V0A2	NM_012463	hydrogen ion transporter activity	-0.49	4.27E-06	9.22	-0.40	2.20E-04	6.67
92	SCML2	NM_006089	transcription factor activity	-0.49	4.59E-06	9.13	-0.44	9.13E-05	7.89
93	ATP11A	NM_015205	magnesium ion binding;ATPase activity	-0.48	5.19E-06	8.99	-0.31	2.40E-03	3.32
94	XPO7	NM_015024	nuclear export signal receptor activity	-0.48	5.33E-06	8.96	-0.47	3.60E-05	9.18
95	ABCA2	NM_001606	ATPase activity	-0.47	5.41E-06	8.94	-0.29	4.57E-03	2.47
96	TAF1	NM_138923	protein serine/threonine kinase activity	-0.46	5.78E-06	8.85	-0.35	5.45E-04	5.38
97	PAPSS2	NM_004670	nucleotide binding;ATP binding	-0.46	7.18E-06	8.58	-0.27	8.58E-03	1.64
98	STX2	NM_194356	protein transporter activity	-0.47	7.78E-06	8.48	-0.35	8.33E-04	4.75
99	MIS12	NM_024039	protein binding	-0.43	8.40E-06	8.39	-0.36	3.42E-04	6.01
100	ENDOGL1	NM_005107	endonuclease activity;hydrolase activity	-0.45	9.50E-06	8.24	-0.38	3.16E-04	6.12
101	EIF4B	NM_001417	RNA binding;translation initiation factor activity	-0.44	9.51E-06	8.24	-0.44	6.14E-05	8.45
102	RBM3	NM_006743	nucleotide binding;RNA binding	-0.48	1.30E-05	7.86	-0.45	1.21E-04	7.51
103	TANC2	XM_371074	binding	-0.41	1.35E-05	7.82	-0.40	1.05E-04	7.71
104	MIS12	NM_024039	protein binding	-0.42	1.36E-05	7.81	-0.47	2.51E-05	9.68
105	FAM40B	NM_020704	hypothetical protein	-0.52	1.62E-05	7.59	-0.69	3.42E-06	12.29
106	SEL1L	NM_005065	binding	-0.43	1.83E-05	7.44	-0.26	1.04E-02	1.39
107	SFXN1	NM_022754	protein binding;cation transporter activity	-0.45	1.90E-05	7.39	-0.72	3.87E-07	15.24
108	TAF11	NM_005643	RNA polymerase II transcription factor activity	-0.40	1.97E-05	7.35	-0.65	3.38E-07	15.48
109	PTPN21	NM_007039	protein tyrosine phosphatase activity	-0.39	2.35E-05	7.14	-0.35	2.92E-04	6.26
110	LIN7C	NM_018362	protein binding	-0.38	2.69E-05	6.97	-0.36	2.34E-04	6.57
111	KBTBD6	NM_152903	protein binding	-0.40	3.05E-05	6.82	-0.37	3.08E-04	6.17
112	E2F7	NM_203394	transcription factor activity	-0.37	3.24E-05	6.75	-0.24	8.83E-03	1.60
113	~	ENSG00000170092	unknown	-0.38	3.58E-05	6.63	-0.36	2.56E-04	6.45
114	SSR3	NM_007107	signal sequence binding;protein binding	-0.43	3.59E-05	6.63	-0.31	3.88E-03	2.68
115	ANKS6	NM_173551	unknown	-0.42	3.60E-05	6.62	-0.43	1.43E-04	7.28
116	ERF	NM_006494	ligand-regulated transcription factor activity	-0.39	3.68E-05	6.60	-0.43	5.21E-05	8.66
117	GSTCD	NM_024751	transferase activity	-0.42	3.83E-05	6.55	-0.42	1.52E-04	7.18
118	MCM4	NM_005914	DNA helicase activity; ATPase activity	-0.39	4.15E-05	6.45	-0.82	3.05E-08	18.61
119	TAF11	NM_005643	RNA polymerase II transcription factor activity	-0.40	4.44E-05	6.37	-0.72	2.30E-07	16.02
120	WDR45L	NM_019613	unknown	-0.38	4.75E-05	6.29	-0.47	2.49E-05	9.70
121	UBQLN1	NM_013438	kinase binding	-0.36	4.88E-05	6.26	-0.44	2.57E-05	9.63
122	STOM	NM_004099	integral to plasma membrane	-0.43	5.00E-05	6.23	-0.93	3.10E-08	18.52
123	DISP1	NM_032890	unknown	-0.35	5.17E-05	6.19	-0.31	8.48E-04	4.73
124	C9orf97	NM_139246	unknown	-0.38	5.23E-05	6.17	-0.28	3.67E-03	2.75
125	USP28	NM_020886	ubiquitin thiolesterase activity	-0.40	5.55E-05	6.10	-0.44	9.58E-05	7.83
126	LIN7C	NM_018362	protein binding	-0.39	5.70E-05	6.06	-0.39	2.44E-04	6.51
127	PTPN14	NM_005401	protein tyrosine phosphatase activity	-0.34	5.80E-05	6.04	-0.26	3.07E-03	2.99
128	FXC1	NM_012192	zinc ion binding	-0.40	5.99E-05	6.00	-0.43	1.32E-04	7.39
129	ASCC3	NM_006828	ATP-dependent helicase activity	-0.45	6.21E-05	5.95	-0.33	4.73E-03	2.42
130	SDF4	NM_016176	calcium ion binding	-0.38	6.65E-05	5.88	-0.30	2.78E-03	3.12
131	ORAOV1	NM_153451	unknown	-0.44	6.72E-05	5.86	-0.57	2.15E-05	9.93
132	NHN1	NM_144604	nucleic acid binding;zinc ion binding	-0.43	7.09E-05	5.80	-0.46	1.52E-04	7.18
133	~	ENSG00000185636	unknown	-0.39	7.48E-05	5.74	-0.39	2.99E-04	6.23
134	SLC17A5	NM_012434	transporter activity	-0.39	7.75E-05	5.69	-0.38	3.56E-04	5.94
135	RABL3	NM_173825	transcription factor binding	-0.36	8.04E-05	5.65	-0.27	4.34E-03	2.53
136	TRPV1	NM_018727	ion transport	-0.37	8.40E-05	5.60	-0.31	1.70E-03	3.80
137	PRR11	NM_018304	unknown	-0.37	8.50E-05	5.58	-0.59	1.94E-06	13.03
138	NCOA4	NM_005437	regulation of transcription	-0.37	8.75E-05	5.55	-0.38	2.95E-04	6.24
139	TMTC3	NM_181783	binding	-0.35	1.16E-04	5.21	-0.35	4.58E-04	5.61

140	FLJ40142	NM_207435	unknown	-0.42	1.19E-04	5.18	-0.40	7.37E-04	4.93
141	EML2	NM_012155	protein binding	-0.35	1.30E-04	5.08	-0.34	6.60E-04	5.10
142	CLSPN	NM_022111	unknown	-0.37	1.39E-04	5.00	-0.47	4.61E-05	8.83
143	SIX4	NM_017420	transcription factor activity	-0.36	1.40E-04	4.99	-0.29	4.75E-03	2.42
144	GSTCD	NM_024751	unknown	-0.40	1.48E-04	4.93	-0.46	1.41E-04	7.30
145	CREB3L2	NM_194071	transcription factor activity	-0.35	1.49E-04	4.91	-0.63	7.97E-07	14.30
146	CLASP1	NM_015282	kinetochore binding;microtubule binding	-0.40	1.90E-04	4.63	-0.35	2.48E-03	3.28
147	~	ENSG00000196393	unknown	-0.35	2.38E-04	4.35	-0.29	4.10E-03	2.61
148	~	ENSG00000203499	unknown	-0.35	2.62E-04	4.24	-0.37	6.08E-04	5.22
149	COX1	NP_536845	cytochrome c oxidase activity	-0.41	2.72E-04	4.19	-1.29	1.38E-09	22.23
150	PSMD2	NM_002808	protein binding	-0.50	2.73E-04	4.29	-0.76	4.51E-04	5.86
151	~	XR_015437.1	unknown	-0.34	2.92E-04	4.11	-0.42	1.20E-04	7.54
152	TSC22D3	NM_198057	transcription factor activity	-0.44	3.26E-04	3.98	-0.51	2.25E-04	6.63
153	FOXJ3	NM_014947	transcription factor activity	-0.31	3.38E-04	3.94	-0.33	5.33E-04	5.42
154	LRRC23	NM_201650	protein binding	-0.32	3.69E-04	3.83	-0.52	6.86E-06	11.37
155	~	ENSG00000197239	unknown	-0.37	3.81E-04	3.79	-0.37	1.25E-03	4.21
156	RBPS2	NM_194272	nucleotide binding	-0.31	3.82E-04	3.79	-0.49	1.30E-05	10.59
157	ROBO1	NM_002941	unknown	-0.38	3.89E-04	3.77	-0.39	1.04E-03	4.46
158	SLC30A6	NM_017964	zinc ion transporter activity	-0.34	4.49E-04	3.59	-0.43	1.35E-04	7.36
159	PXK	NM_017771	actin binding;phosphoinositide binding	-0.30	4.78E-04	3.52	-0.44	3.38E-05	9.28
160	ZBT34	XM_928961.2	regulation of transcription	-0.29	5.09E-04	3.45	-0.49	5.07E-06	11.80
161	USP33	NM_015017	ubiquitin thiolesterase activity	-0.29	5.12E-04	3.44	-0.35	2.86E-04	6.30
162	CXorf34	NM_024917	methyltransferase activity	-0.40	5.64E-04	3.33	-0.40	1.95E-03	3.60
163	ZBTB24	NM_014797	DNA binding;protein binding	-0.37	5.80E-04	3.29	-0.39	1.22E-03	4.25
164	CARD14	NM_024110	protein binding;CARD domain binding	-0.35	5.95E-04	3.26	-0.36	1.36E-03	4.10
165	HIST1H2AG	NM_021064	DNA binding	-0.31	6.14E-04	3.32	-0.39	6.05E-03	2.39
166	FANCE	NM_021922	protein binding	-0.29	6.41E-04	3.17	-0.29	2.11E-03	3.50
167	PPP2R5B	NM_006244	protein phosphatase type 2A regulator activity	-0.29	6.51E-04	3.15	-0.33	5.41E-04	5.39
168	BDP1	NM_018429	DNA binding;translation initiation factor activity	-0.31	6.91E-04	3.08	-0.34	1.03E-03	4.47
169	ACBD5	NM_145698	acyl-CoA binding;binding	-0.33	6.99E-04	3.07	-0.45	1.14E-04	7.60
170	B2L13	NM_015367	induction of apoptosis	-0.38	7.02E-04	3.06	-0.36	3.31E-03	2.89
171	KIAA1922	XM_057040	unknown	-0.34	7.23E-04	3.03	-0.36	9.78E-04	4.55
172	RANBP2	NM_006267	peptidyl-prolyl cis-trans isomerase activity	-0.33	7.38E-04	3.00	-0.46	7.07E-05	8.26
173	CDC14A	NM_033313	mitotic spindel regulation activity	-0.31	7.85E-04	2.93	-0.68	4.85E-07	15.00
174	WNT5B	NM_030775	signal transducer activity;protein binding	-0.33	7.89E-04	2.93	-0.36	8.34E-04	4.75
175	FKBP14	NM_017946	peptidyl-prolyl cis-trans isomerase activity	-0.38	7.94E-04	2.92	-0.54	8.29E-05	8.02
176	ANGEL2	NM_144567	hypothetical protein	-0.32	8.35E-04	2.85	-0.41	2.44E-04	6.51
177	EEF1D	NM_032378	translation elongation factor activity	-0.30	8.44E-04	2.84	-0.28	4.27E-03	2.55
178	CXorf56	NP_071384	protein binding	-0.36	8.84E-04	2.79	-0.97	4.07E-08	18.01
179	ZNF430	NM_025189	DNA binding;zinc ion binding	-0.29	8.91E-04	2.78	-0.40	1.14E-04	7.60
180	Q6A1A2	ENST00000382326	kinase activity	-0.42	9.26E-04	2.73	-0.39	5.35E-03	2.25
181	FAM73A	NM_198549	unknown	-0.32	9.53E-04	2.70	-0.28	9.11E-03	1.56
182	CYP51A1	NM_000786	monooxygenase activity;iron ion binding	-0.29	9.62E-04	2.69	-0.62	8.18E-07	14.22
183	LOC390414	XR_015582	unknown	-0.32	9.67E-04	2.68	-0.40	3.42E-04	6.02
184	PPP2R5C	NM_002719	protein phosphatase type 2A regulator activity	-0.31	1.02E-03	2.62	-0.36	5.53E-04	5.36
185	C20orf177	NM_022106	unknown	-0.29	1.23E-03	2.40	-0.29	3.31E-03	2.89
186	HEAT1	NM_018072	protein binding	-0.34	1.25E-03	2.38	-0.60	1.11E-05	10.79
187	~	ENSG00000196548	unknown	-0.31	1.33E-03	2.31	-0.42	2.17E-04	6.68
188	~	ENSG00000197187	unknown	-0.26	1.54E-03	2.13	-0.38	1.14E-04	7.60
189	HIAL2	NR_002894	transporter activity	-0.29	1.68E-03	2.04	-0.34	8.53E-04	4.72
190	TBP	NM_003194	RNA polymerase II transcription factor activity	-0.74	1.76E-03	2.42	-0.68	2.32E-03	3.62
191	GTF2H3	NM_001516	transcription factor activity	-0.33	1.88E-03	1.90	-0.41	5.98E-04	5.24
192	UTY	NM_182660	unknown	-0.29	1.91E-03	1.88	-0.30	3.46E-03	2.83
193	SNX12	NM_013346	sorting nexin family	-0.30	2.03E-03	1.81	-0.51	2.56E-05	9.64
194	Q8WUS3	ENSG00000198596	unknown	-0.31	2.12E-03	1.76	-0.35	1.53E-03	3.94
195	TNKS1BP1	NM_033396	enzyme binding;ankyrin binding	-0.36	2.25E-03	1.68	-0.37	4.39E-03	2.52
196	RIR2	NM_001034	DNA replication	-0.28	2.28E-03	1.67	-0.40	1.85E-04	6.90
197	SMC5	NM_015110.2	DNA repair	-0.27	2.29E-03	1.66	-0.34	5.45E-04	5.38
198	SS18	NM_005637	unknown	-0.40	2.31E-03	1.65	-0.40	5.46E-03	2.22
199	~	ENSG00000198069	unknown	-0.30	2.36E-03	1.63	-0.40	4.36E-04	5.68
200	LATS1	NM_004690	protein serine/threonine kinase activity	-0.27	2.48E-03	1.57	-0.28	4.15E-03	2.59
201	UTX	NM_021140	binding	-0.25	2.59E-03	1.52	-0.25	6.37E-03	2.03
202	AURKA	NM_198433	protein serine/threonine kinase activity	-0.30	2.62E-03	1.50	-0.33	2.50E-03	3.27
203	ARNT	NM_001668	signal transduction	-0.28	2.80E-03	1.43	-0.38	4.39E-04	5.67
204	ARID1A	NM_006015	transcriptional activator activity	-0.25	2.82E-03	1.42	-0.77	3.65E-08	18.27
205	PIP5K1A	NM_003557	kinase activity	-0.37	2.86E-03	1.41	-0.66	2.04E-05	9.99
206	UMPS	NM_000373	transferase activity	-0.30	2.88E-03	1.40	-0.34	2.69E-03	3.17
207	VIPR2	NM_003382	receptor activity	-0.45	3.40E-03	1.45	-0.46	6.52E-03	2.20
