# Supplementary material for: Off-target effects of siRNA specific for GFP
Source: BMC Mol Biol. 2008 Jun 24;9:60. doi: 10.1186/1471-2199-9-60 (PMC2443166; doi:10.1186/1471-2199-9-60)
Supplement: Additional file 3 — mRNAs deregulated by GFP siRNA contain short sequences perfectly matched to the sense or antisense strand of GFP siRNA. In order to define the background level of homologies to short sequence motifs, we i) shuffled the GFP siRNA sequence and ii) searched for homologies in genes that were either not deregulated or randomly picked. Shuffling of the GFP siRNA sequence was performed either with preserving the dinucleotide composition or without preservation of the dinucleotide composition (see Methods for details). The resulting shuffled GFP siRNA sequence was aligned to the set of genes that were deregulated by the proper GFP siRNA sequence in the profiling experiment ("shuffled dinucleotides preserved" and "shuffled dinucleotides not preserved"). For the negative control set of non-deregulated genes, we selected 207 genes that were least deregulated after transfection of GFP siRNA ("not dereg.A" and "not dereg. B") and 207 genes that were least deregulated in an unrelated profiling experiment ("diff.array", for details see Methods). We scored motifs of different lengths (ranging from >7 bp to >10 bp) that were homologous to the sequence of the transfected GFP siRNA. When comparing the sequence of GFP siRNA to sequences of the 207 significantly down regulated genes (grey bar), more genes with homology were found than in two sets of genes that were not regulated ("not dereg.A"; "not dereg.B") or taken from a non-related array ("diff.array"). With increasing length of homology, relatively more hits were scored for the proper, non-shuffled GFP siRNA in the set of actually deregulated genes as compared to all negative controls (bottom panels). [file 1471-2199-9-60-S3.pdf]

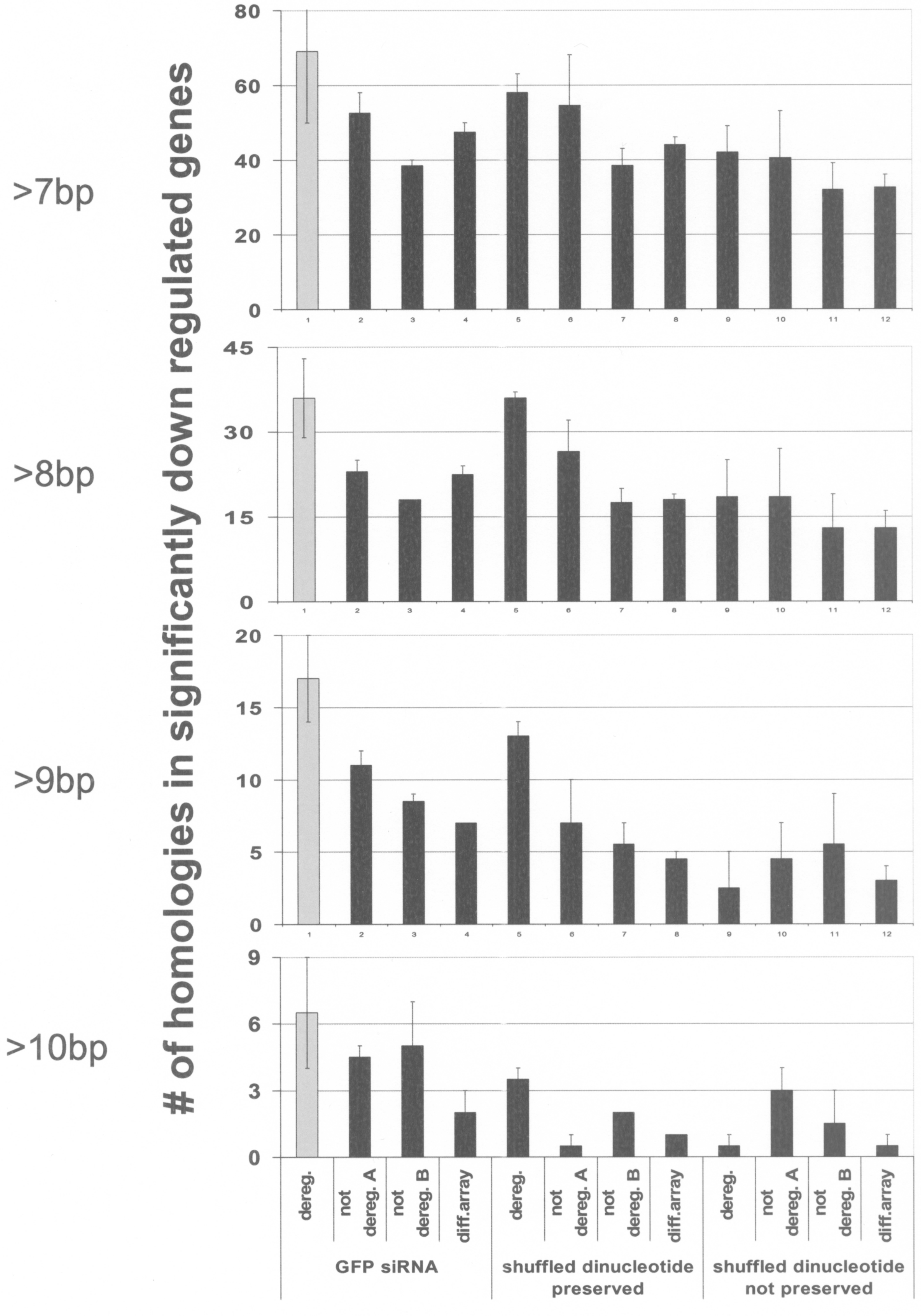

>7bp
>8bp
>9bp
>10bp
# of homologies in significantly down regulated genes
80
60
40
20
0
45
30
15
0
20
15
10
5
0
9
6
3
0
1
2
3
4
5
6
7
8
9
10
11
12
dereg.
not dereg. A
not dereg. B
diff.array
GFP siRNA
shuffled dinucleotide preserved
shuffled dinucleotide not preserved
