# Supplementary material for: Off-target effects of siRNA specific for GFP
Source: BMC Mol Biol. 2008 Jun 24;9:60. doi: 10.1186/1471-2199-9-60 (PMC2443166; doi:10.1186/1471-2199-9-60)
Supplement: Additional file 5 — Description of normalization of microarray data. [file 1471-2199-9-60-S5.pdf]

Additional file 5

After normalization, a linear model was fit to the log2 expression ratios according to the specific experiments. A common reference design for the cell lines HELA and HEK was employed that used dye swaps and two replicates of each experiment resulting in 4 technical replicates each. The design matrix is given by the table below: '-1' indicates that the cell line was labelled with Cy3 and '1' that the cell line was labelled with Cy5 in the common reference design.

HELA	HEK
-1	0
1	0
-1	0
1	0
0	-1
0	1
0	-1
0	1

Empirical Bayes was used as shrinkage procedure to moderate the standard deviations between genes providing a moderated t statistic [1]. P value adjustment was performed according to Benjamini and Yekutieli. An adjusted p value lower than 5% and the probability of being differentially expressed of at least 0.98 was the necessary condition for being selected. The B statistic is the log-posterior odds of differential expression. Suppose that B = 4, the odds of differential expression, are then given by: e^B. The probability that the gene is differentially expressed can then be computed as $e^B/ (1+e^B) = 0.98$, which means the probability is about 98% that this gene is differentially expressed. The B statistics is adjusted for multiple testing by assuming that 1% of the genes are expected to be differentially expressed.

1. Smyth GK: **Linear models and empirical bayes methods for assessing differential expression in microarray experiments**. *Stat Appl Genet Mol Biol* 2004, **3**:Article3.
