# Supplementary material for: Off-target effects of siRNA specific for GFP
Source: BMC Mol Biol. 2008 Jun 24;9:60. doi: 10.1186/1471-2199-9-60 (PMC2443166; doi:10.1186/1471-2199-9-60)
Supplement: Additional file 4 — qPCR primer sequences. [file 1471-2199-9-60-S4.pdf]

Additional file 4: qPCR primer sequences.			
**gene**	**RefSeq**	**forward**	**reverse**
CYLD	NM_015247	gtcttctgttgactcacaaccac	acggtcaaagtctgtagatatctc
SOAT	NM_003101	ctatgcttacaaggactttctctg	caaggcatattcgtgtactacag
PGK	NM_000291	aagtgaagctcggaaagcttctat	tgggaaaagatgcttctggg
DCTN2	NM_006400	actagcgacctacctgagga	tcataggcagcattaggattgac
LMNB1	NM_005573	ctggaaatgtttgcatcgaaga	gcctcccattggttgatcc
C13ORF1	NM_020456	ccctttaccacaacaatgaagaga	tacctgatgctggacaatgcatg
OAS1	NM_016816	caagagcctcatccgcctag	tgctccctcgctcccaag
